# CENPN suppresses autophagy and increases paclitaxel resistance in nasopharyngeal carcinoma cells by inhibiting the CREB-VAMP8 signaling axis

**DOI:** 10.1080/15548627.2023.2258052

**Published:** 2024-01-25

**Authors:** Bin-Ru Wang, Ji-Bo Han, Yang Jiang, Shan Xu, Rui Yang, Yong-Gang Kong, Ze-Zhang Tao, Qing-Quan Hua, You Zou, Shi-Ming Chen

**Affiliations:** a Department of Otolaryngology-Head and Neck Surgery, Renmin Hospital of Wuhan University, Wuhan, Hubei, P.R. China; b Institute of Otolaryngology-Head and Neck Surgery, Renmin Hospital of Wuhan University, Wuhan, Hubei, P.R. China

**Keywords:** Autophagy, CENPN, chemotherapeutic resistance, nasopharyngeal carcinoma, paclitaxel

## Abstract

Chemotherapeutic resistance is one of the most common reasons for poor prognosis of patients with nasopharyngeal carcinoma (NPC). We found that CENPN can promote the growth, proliferation and apoptosis resistance of NPC cells, but its relationship with chemotherapeutic resistance in NPC is unclear. Here we verified that the CENPN expression level in NPC patients was positively correlated with the degree of paclitaxel (PTX) resistance and a poor prognosis through analysis of clinical cases. VAMP8 expression was significantly increased after knockdown of *CENPN* by transcriptome sequencing. We found in cell experiments that CENPN inhibited macroautophagy/autophagy and VAMP8 expression and significantly increased PTX resistance. Overexpression of *CENPN* reduced the inhibitory effects of PTX on survival, cell proliferation, cell cycle progression and apoptosis resistance in NPC cells by inhibiting autophagy. In turn, knockdown of *CENPN* can affect the phenotype of NPC cells by increasing autophagy to achieve PTX sensitization. Sequential knockdown of *CENPN* and *VAMP8* reversed the PTX-sensitizing effect of *CENPN* knockdown alone. Experiments in nude mice confirmed that knockdown of *CENPN* can increase VAMP8 expression, enhance autophagy and increase the sensitivity of NPC cells to PTX. Mechanistic studies showed that CENPN inhibited the translocation of p-CREB into the nucleus of NPC cells, resulting in the decreased binding of p-CREB to the *VAMP8* promoter, thereby inhibiting the transcription of *VAMP8*. These results demonstrate that CENPN may be a marker for predicting chemotherapeutic efficacy and a potential target for inducing chemosensitization to agents such as PTX.

**Abbreviations:** 3-MA: 3-methyladenine; ATG5: autophagy related 5; CENPN: centromere protein N; CQ: chloroquine; CREB: cAMP responsive element binding protein; ChIP: chromatin immunoprecipitation assay; IC50: half-maximal inhibitory concentration; LAMP2A: lysosomal associated membrane protein 2A; MAP1LC3/LC3: microtubule associated protein 1 light chain 3; NPC: nasopharyngeal carcinoma; NPG: nasopharyngitis; oe*CENPN*: overexpressed *CENPN*; PTX: paclitaxel; RAPA: rapamycin; RNA-seq: transcriptome sequencing; sh*CENPN*: small hairpin RNA expression vector targeting the human *CENPN* gene; sh*CENPN*-sh*VAMP8*: sequential knockdown targeting the human *CENPN* gene and *VAMP8* gene; sh*VAMP8*: small hairpin RNA expression vector targeting the human *VAMP8* gene; TEM: transmission electron microscopy; TIR: tumor inhibitory rate; VAMP8: vesicle associated membrane protein 8.

## Introduction

Due to the occult pathogenesis and the atypical early symptoms of nasopharyngeal carcinoma (NPC), more than 70% of patients are diagnosed in middle and advanced stages [1,2]. Intensity-modulated radiotherapy combined with chemotherapy can significantly improve the survival of patients with advanced NPC, but even so, the 5-year survival rate is still less than 20% [3,4]. Chemotherapeutic resistance is one of the most common reasons for the poor prognosis and low survival rate of patients with various cancers (including NPC), and it is the main factor that limits the clinical application of chemotherapeutic drugs [5].

Macroautophagy/autophagy is a crucial mechanism for the maintenance of cellular and organismal homeostasis [6,7]. Autophagy plays an important role in inhibiting malignant transformation, cancer progression and reducing chemotherapy resistance through autophagy-dependent cell death, autosis, ferroptosis, and other pathways [8-10]. Autophagy-dependent cell death is a unique programmed cell death mechanism, which is not associated with apoptosis or necroptosis [11]. Compared with self-protective autophagy, autophagy-dependent cell death induced by chemotherapeutic drugs such as paclitaxel (PTX) plays a self-destructive role in cancer cells and thus greatly enhances the efficacy of chemotherapeutic drugs, which is one of the prominent research directions in the effort to overcome chemotherapeutic resistance [12].

Relevant studies have shown that inhibition of autophagy will lead to the increase of malignant degree such as growth and metastasis of NPC, and chemotherapeutic drugs can enhance the chemotherapy sensitivity of NPC by activating autophagy [13]. Many factors play important roles in the dynamic process of cell metabolism in the multi-stage process of autophagy. ATG5 (autophagy related 5) plays an important role in regulating the initiation and formation of autophagosomes [14]. LAMP2A (lysosomal associated membrane protein 2A), an important component of the lysosomal membrane, plays an important role in the formation of lysosomes and is also involved in the phagocytosis and the late stage of autophagy. LAMP2A could not only promote the level of ATG5 in the brain of Drosophila, but also prevent the accumulation of SQSTM1/p62 (sequestosome 1), thus activating autophagic flux (i.e., the rate of protein degradation through the entire autophagy pathway) and improving the resistance of Drosophila brain cells to oxidative stress [15,16]. When autophagy is activated, the conversion of LC3-I to LC3-II increases, the number of autophagosomes and autolysosomes increases, and the level of the autophagic degradation substrate SQSTM1 decreases [17].

PTX is one of the standard chemotherapeutic drugs for head and neck squamous cell carcinoma, lung cancer, ovarian cancer, breast cancer and other cancers and is also used in first-line treatment of NPC [18-20]. However, approximately 30% of patients with advanced NPC develop chemotherapeutic resistance and distant metastasis after PTX treatment, and the mechanism is still unclear [21,22]. Delineating the resistance mechanism of PTX and exploring sensitization strategies are of great significance for improving the clinical outcomes of patients with advanced NPC.

CENPN (centromere protein N) recognizes the N-terminal region of the CENPA nucleosome and binds to CENPL to form a mitophagy-associated network [23]. Our previous study showed that CENPN can promote the proliferation and apoptosis resistance of NPC cells and is closely related to the prognosis of NPC patients [24]. Chemotherapy is an important means of NPC treatment; oncogenes can confer chemotherapeutic resistance, but whether CENPN also affects the prognosis of NPC patients by increasing chemotherapeutic resistance remains unclear.

CREB (cAMP regulatory element binding protein) is an important nuclear transcription factor belonging to the basic leucine zipper (bZIP) family [25]. CREB and phospho-CREB (p-CREB) are key gene products involved in the proliferation, apoptosis and metastasis of cancer cells such as lung cancer and glioma cells, as well as important factors affecting the chemotherapy resistance of tumors [26-28]. However, the relationship between p-CREB and the chemotherapy sensitivity of NPC has not been reported.

VAMP8 (vesicle associated membrane protein 8) plays a decisive role in the regulation of lysosome-autophagosome association and lysosomal aggregation, and inhibition of VAMP8 can block the process of autophagy [29,30]. VAMP8 often acts as a “switch” between autophagosome membrane contact and fusion to control changes in autophagic flux [31]. Our previous studies found that knockdown of *CENPN* in NPC cells significantly increased *VAMP8* expression; thus, it was speculated that CENPN may affect chemoresistance in NPC through regulation of VAMP8 expression and autophagy. This study explored the molecular mechanism of CENPN-induced PTX resistance and poor survival in NPC through in vitro and in vivo experiments, aiming to provide an experimental reference for sensitizing NPC to chemotherapy.

## Results

### CENPN is closely related to PTX resistance and poor prognosis in patients with NPC and is negatively correlated with VAMP8 expression and autophagy levels

**Figure 1.**
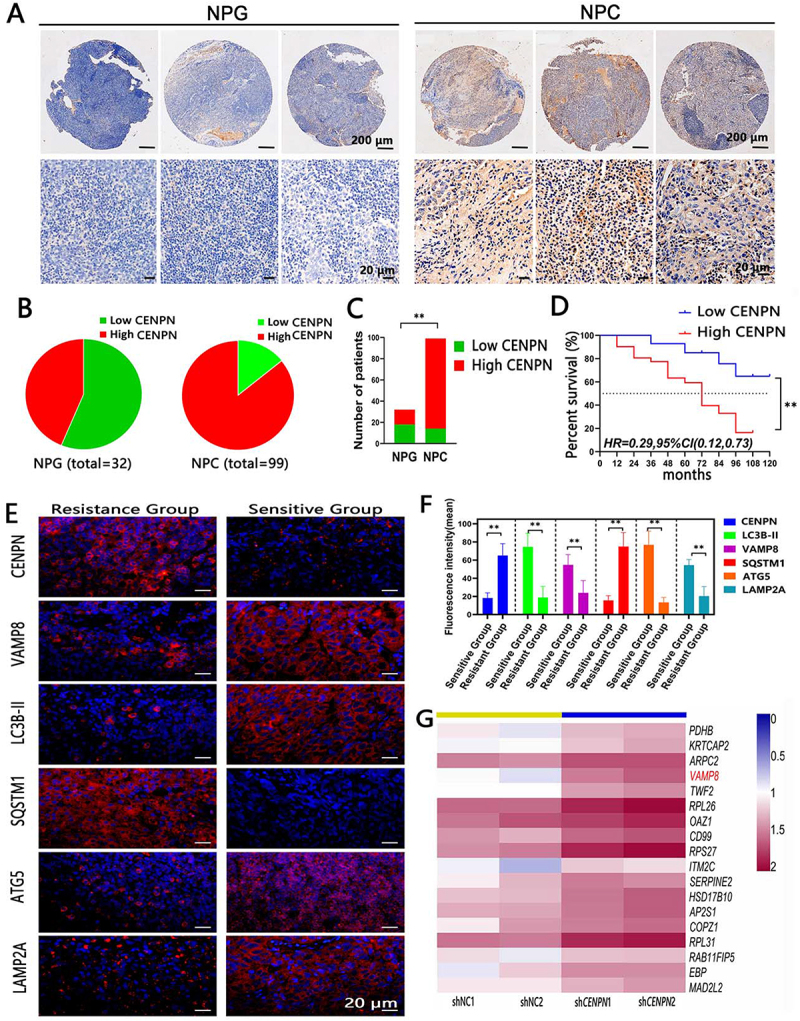
Expression of CENPN in NPC tissues is closely related to autophagy and poor prognosis. (A) Representative immunohistochemical images showing the differential expression of CENPN in nasopharyngitis (NPG) and NPC (50× and 400×). (B, C) Immunohistochemical scoring results. (D) Survival curves of NPC patients stratified by the CENPN expression level. (E, F) Representative immunofluorescence images showing the differential protein levels of CENPN, VAMP8, LC3B-II, SQSTM1, ATG5 and LAMP2A in patients with paclitaxel-resistant and paclitaxel-sensitive NPC (400×). (G) RNA-seq identification of upregulated genes after inhibition of *CENPN*. Data are presented as mean ± SD. **, P<0.01.

Immunohistochemical scoring of the tissue microarray (TMA) showed that the expression level of CENPN in the 99 NPC patients was significantly higher than that in the 32 nasopharyngitis (NPG) patients (Figure 1A-C). In addition, survival analysis of the 45 NPC patients with 10-year follow-up data showed that the prognosis of patients with high CENPN expression was significantly worse than that of patients with low CENPN expression (Figure 1D).

In the following analysis of PTX efficacy in 35 NPC patients, there were no significant differences in general oncological characteristics between the chemotherapy-sensitive and chemotherapy-resistant groups (**Table S1**.). The immunofluorescence scores showed that CENPN expression was significantly higher in the chemotherapy-resistant group than in the chemotherapy-sensitive group (Figure 1E-F).

The transcriptome sequencing (RNA-seq) heatmap showed that the expression of *VAMP8* was significantly increased after knockdown of *CENPN* in CNE-2Z cells (Figure 1G).

Since the expression of VAMP8 is related to autophagy, we evaluated the levels of the LC3-II, VAMP8, SQSTM1, LAMP2A and ATG5 in clinical specimens from 35 NPC patients undergoing PTX chemotherapy. The results showed that in the chemotherapy-sensitive group, the LC3B-II, VAMP8, LAMP2A and ATG5 levels were significantly higher than those in the chemotherapy-resistant group, and the levels of CENPN and SQSTM1 were significantly lower than those in the chemotherapy-resistant group (Figure 1F). CENPN exhibited a negative correlation with VAMP8, LC3-II, LAMP2A and ATG5 and a positive correlation with SQSTM1. VAMP8, LC3B-II, LAMP2A and ATG5 were positively correlated with each other, and all were negatively correlated with SQSTM1 (Fig. S1A).

### CENPN expression induced significant PTX resistance and changes in autophagy levels in NPC cells in vitro

**Figure 2.**
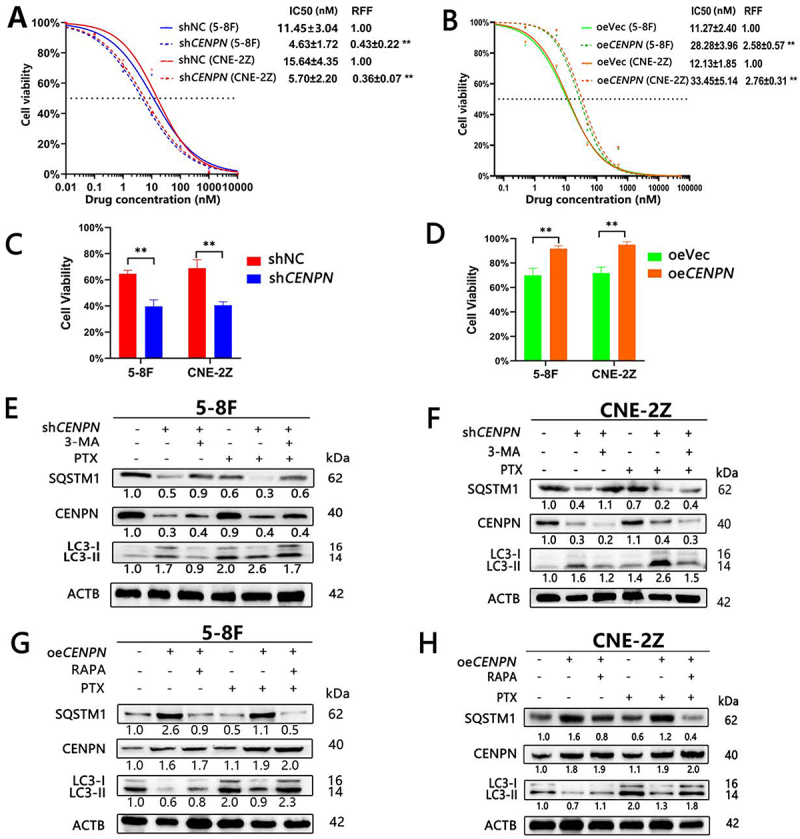
CENPN expression affects autophagy and PTX resistance in NPC cells. (A) The cytotoxicity assay showed that knockdown of *CENPN* reduced PTX resistance in NPC cells. (B) The cytotoxicity assay showed that overexpression of *CENPN* enhanced PTX resistance in NPC cells. (C) The CCK8 assay showed the effect of PTX (10 nM) on the viability of NPC cells after knockdown of *CENPN*. (D) The CCK8 assay showed the effect of PTX (5 nM) on the viability of NPC cells after overexpression of *CENPN*. (E, F) WB analysis showed the effect of PTX (10 nM) on the levels of autophagy in NPC cells after knockdown of *CENPN*. (G, H) WB analysis showed the effect of PTX (5 nM) on the levels of autophagy in NPC cells after overexpression of *CENPN*. Data are presented as mean ± SD.*, P<0.05. **, P<0.01.

In 5-8F and CNE-2Z cells, the IC50 of PTX in the small hairpin RNA expression vector targeting human *CENPN* gene (sh*CENPN*) group was significantly decreased compared with that in the negative control with small hairpin RNA expression vector (shNC) group, and the RRF in the sh*CENPN* group was decreased by 60% and 64%, respectively (Figure 2A). The IC50 of PTX in the overexpression *CENPN* (oe*CENPN*) group was significantly increased compared with that in the over-expression vector (oeVec) group, and the RRF in the oeCENPN group was increased by 151% and 176%, respectively (Figure 2B).

In 5-8F and CNE-2Z cells, when the PTX concentration was 10 nM, cell viability in the sh*CENPN* group (39.67±5.03%, 40.57 ±2.61%, respectively) was significantly lower than that in the shNC group (64.66±3.97%, 68.87±6.54%, respectively) (Figure 2C). When the PTX concentration was 5 nM, cell viability in the oe*CENPN* group (91.71±2.36%, 94.97±2.38%, respectively) was significantly higher than that in the oeVec group (71.17±5.87%, 71.77±4.75%, respectively) (Figure 2D).

Compared with that in the shNC group, the level of autophagy was increased in the sh*CENPN* group (LC3-II:LC3-I, LAMP2A and ATG5 were increased, and SQSTM1 was decreased). After treatment with PTX, autophagy in both the shNC group and the sh*CENPN* group was increased, but the increase in the sh*CENPN* group was more significant. However, 3-MA blocked PTX-induced autophagy in the sh*CENPN* group (Figure 2E-F, Fig. S 1B-C).

Compared with that in the oeVec group, autophagy was attenuated in the oe*CENPN* group (the LC3-II:LC3-I, LAMP2A and ATG5 were decreased, and the SQSTM1 was increased). After treatment with PTX, autophagy in both the oeVec group and the oe*CENPN* group was increased, but the increase in the oeVec group was more significant. However, RAPA reversed the reduction in autophagy in the oe*CENPN* group (Figure 2G-H, Fig. S1D-E).

### CENPN expression can significantly inhibit autophagy in NPC cells

**Figure 3.**
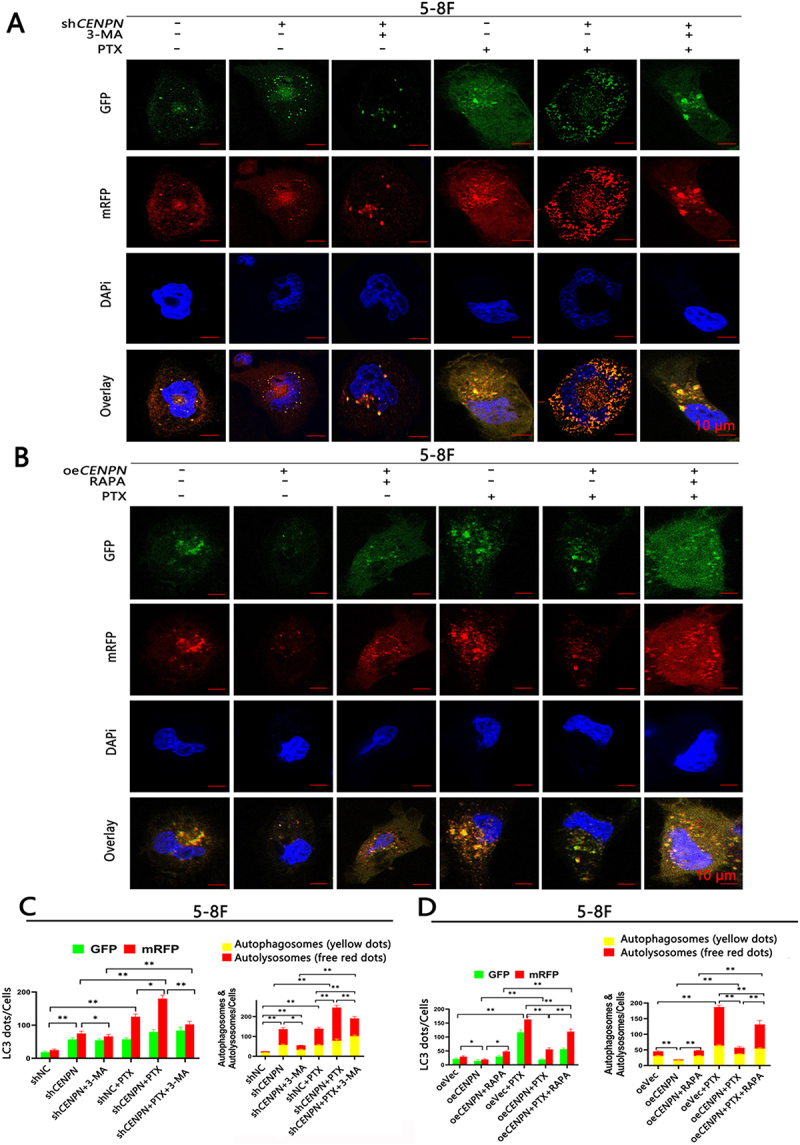
CENPN expression affects PTX-induced autophagic flux in 5-8F cells. (A, C) The tandem mRFP-GFP-LC3 reporter assay showed that the level of autophagic flux in the sh*CENPN*+PTX group was significantly higher than that in the shNC+PTX group. (B, D) The tandem mRFP-GFP-LC3 reporter assay showed that the autophagic flux in the oe*CENPN*+PTX group was significantly lower than that in the oeVec+PTX group. The concentrations of PTX were 10 nM (A, C) and 5 nM (B, D). Data are presented as mean ± SD. *, P<0.05. **, P<0.01.

The tandem mRFP-GFP-LC3 reporter can be used to monitor and quantify changes in intracellular autophagic flux. Compared with the shNC group, the sh*CENPN* group showed increased numbers of autophagosomes and autolysosomes. Compared with the shNC group and sh*CENPN* group, the sh*CENPN*+PTX group showed significantly increased numbers of autophagosomes and autolysosomes, while 3-MA blocked the increase in autophagy induced by sh*CENPN*. Compared with those in the oeVec group, the numbers of intracellular autophagosomes and autolysosomes in the oe*CENPN* group were decreased. Compared with those in the oeVec+PTX group, the numbers of autophagosomes and autolysosomes were significantly decreased in the oe*CENPN*+PTX group, while RAPA reversed the autophagy resistance induced by overexpression of *CENPN* (Figure 3, Fig. S2).

Transmission electron microscopy (TEM) can be used to observe autophagic vacuoles in cells, thus confirming the occurrence of autophagy. Compared with that in the shNC group, the number of autophagosomes in the sh*CENPN* group was increased. Compared with those in the shNC group and sh*CENPN* group, the number of autophagosomes was significantly increased in the sh*CENPN*+PTX group, while 3-MA blocked the increase in autophagy induced by sh*CENPN*. Compared with that in the oeVec group, the number of autophagosomes in the oe*CENPN* group was decreased. Compared with that in the oeVec+PTX group, the number of autophagosomes was significantly decreased in the oe*CENPN*+PTX group, while RAPA reversed the autophagy resistance induced by overexpression of *CENPN* (Fig. S3).

### Inhibition of autophagy by CENPN affects the sensitivity of NPC cells to PTX, as indicated by the effects on survival, clonal proliferation, cell cycle progression and apoptosis

Next, we explored the phenotypic changes in NPC cells after *CENPN* knockdown or *CENPN* overexpression and treatment with PTX at concentrations of 10 nM and 5 nM.

**Figure 4.**
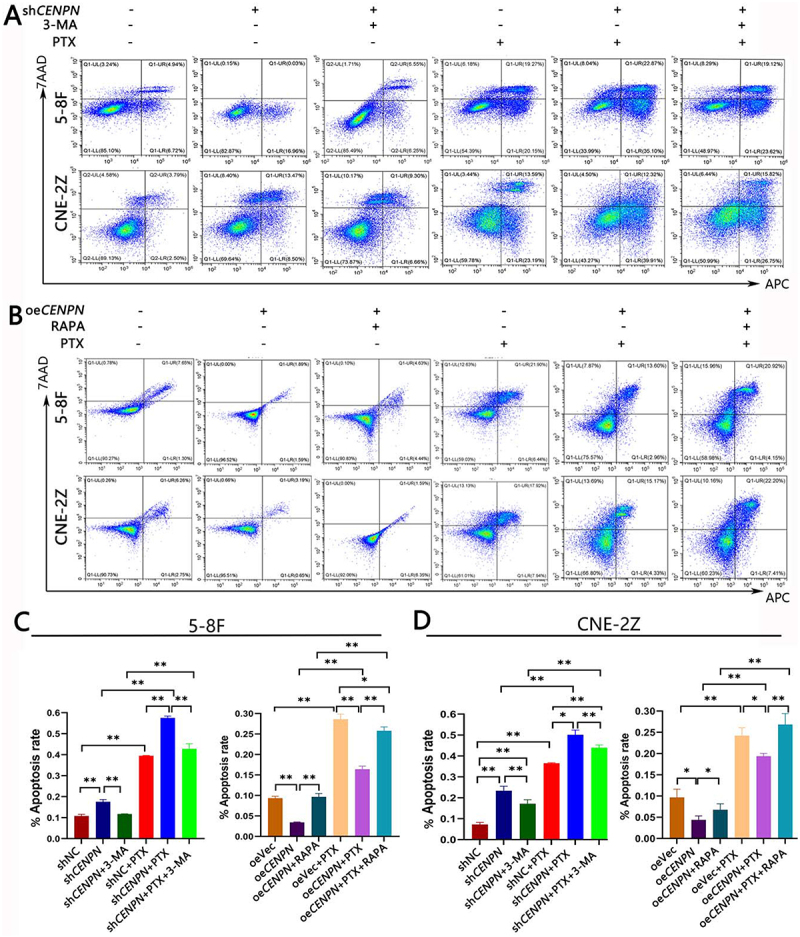
CENPN expression inhibits apoptosis and decreases sensitivity to PTX in NPC cells. (A) Flow cytometry showed the effect of PTX (10 nM) on apoptosis in NPC cells after *CENPN* knockdown. (B) Flow cytometry showed the effect of PTX (5 nM) on apoptosis in NPC cells after *CENPN* overexpression. (C) Bar chart showed the effect of PTX on apoptosis in 5-8F cells after *CENPN* knockdown or *CENPN* overexpression. (D) Bar chart showed the effect of PTX on apoptosis in CNE-2Z cells after *CENPN* knockdown or *CENPN* overexpression. Data are presented as mean ± SD. *, P<0.05. **, P<0.01.

Compared with the sh*CENPN* group, the OD value and clonally proliferation in the sh*CENPN*+PTX group was significantly decreased. Compared with the sh*CENPN*+PTX group, the OD value and clonally proliferation in the sh*CENPN*+PTX+3-MA group was significantly increased (Fig. S4 and Fig. S5). Compared with that in the sh*CENPN* group, the cell cycle arrest and apoptosis in the sh*CENPN*+PTX group was significantly increased. Compared with that in the sh*CENPN*+PTX group, the apoptosis and cell cycle arrest in the sh*CENPN*+PTX+3-MA group was significantly decreased (Figure 4, Fig. S6).

Compared with those in the oeVec+PTX group, the OD value and clonal proliferation in the oe*CENPN*+PTX group were significantly increased. Compared with those in the oe*CENPN*+PTX group, the OD value and clonal proliferation in the oe*CENPN*+PTX+RAPA group were significantly decreased (Fig. S4 and Fig. S5). Compared with that in the oeVec+PTX group, the cell cycle arrest and apoptosis in the oe*CENPN*+PTX group was significantly decreased. Compared with that in the oe*CENPN*+PTX group, the apoptosis and cell cycle arrest in the oe*CENPN*+PTX+RAPA group was significantly increased (Figure 4, Fig. S6).

### *Knockdown of* CENPN *activates autophagy in NPC cells by promoting the fusion of autophagosomes with lysosomes*

**Figure 5.**
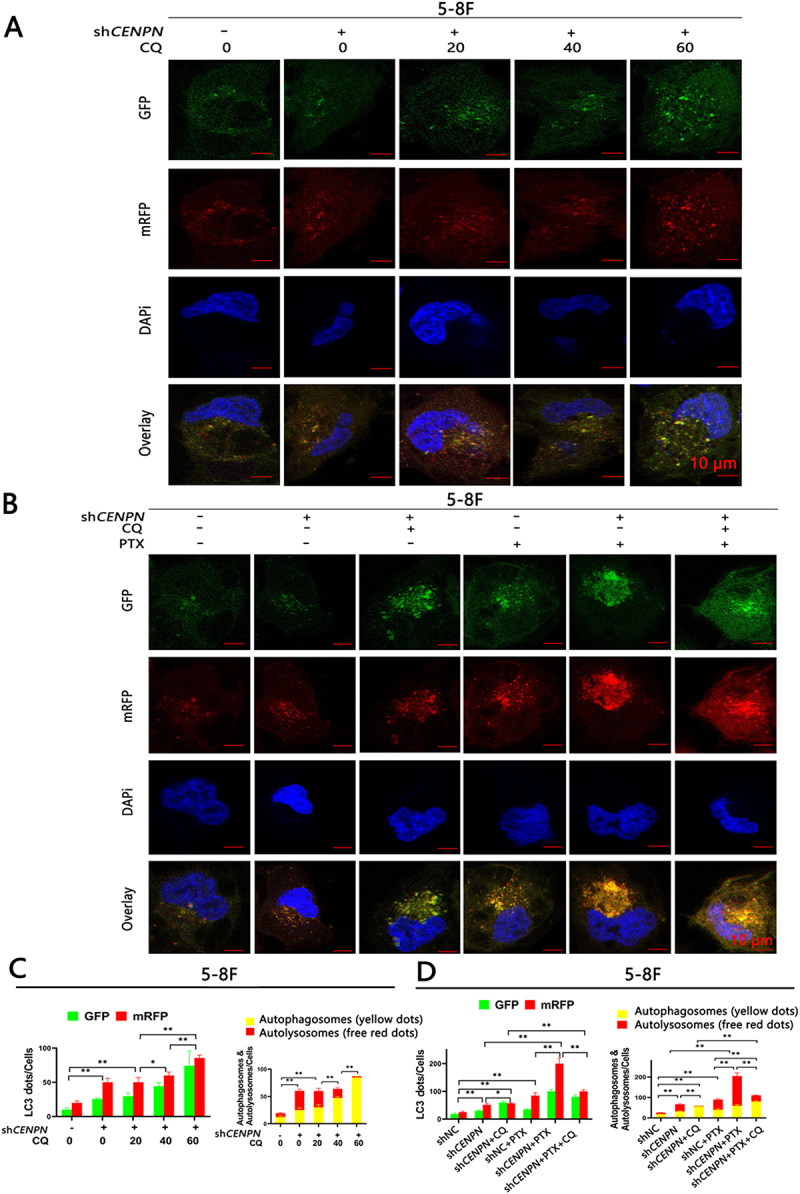
Chloroquine inhibited the activation of autophagic flux in 5-8F sh*CENPN* cells. (A, C) The tandem mRFP-GFP-LC3 reporter assay showed that different concentrations of chloroquine (CQ) could affect the autophagic flux in 5-8F sh*CENPN* cells. (B, D) The tandem mRFP-GFP-LC3 reporter assay showed that the autophagic flux was completely blocked by the saturated concentration of chloroquine (60 μM) in 5-8F sh*CENPN* cells. Data are presented as mean ± SD. *, P<0.05. **, P<0.01.

To further verify that the autophagy level of NPC cells was significantly increased after knockdown of *CENPN*, chloroquine (CQ) was selected to detect autophagic flux in *CENPN*-knockdown 5-8F cells. WB showed that compared with shNC group, LC3-II increased and SQSTM1 decreased in sh*CENPN* group. In 5-8F sh*CENPN* cells, CQ (from10 μM to 40 μM) gradually suppressed SQSTM1 degradation and gradually increased endogenous LC3-II levels, while no further increase of endogenous LC3-II and SQSTM1 was observed when more than 60 μM of CQ was employed (Fig. S7A). The tandem mRFP-GFP-LC3 reporter confirmed that 60 μM of CQ almost completely blocked the autophagic flux in sh*CENPN* cells (Figure 5A,C). These results indicated that 60 μM is the saturated concentration of CQ required to completely block autophagosome-lysosome fusion in sh*CENPN* cells.

The results of autophagic flux test showed that, saturated concentration of CQ (60 μM) could not only completely blocked the enhancement of autophagy induced by *CENPN* knockdown, but also significantly inhibited the increased sensitivity to PTX (Figure 5B,D). Phenotypic experiments showed that, CQ (40 μM) could effectively inhibit the decrease of cell viability, cellular survival, clonal proliferation and PTX sensitivity in *CENPN*-knockdown NPC cells (Fig. S7B-F).

### *Knockdown of* CENPN *reduces PTX resistance in NPC by promoting VAMP8 to activate autophagy*

**Figure 6.**
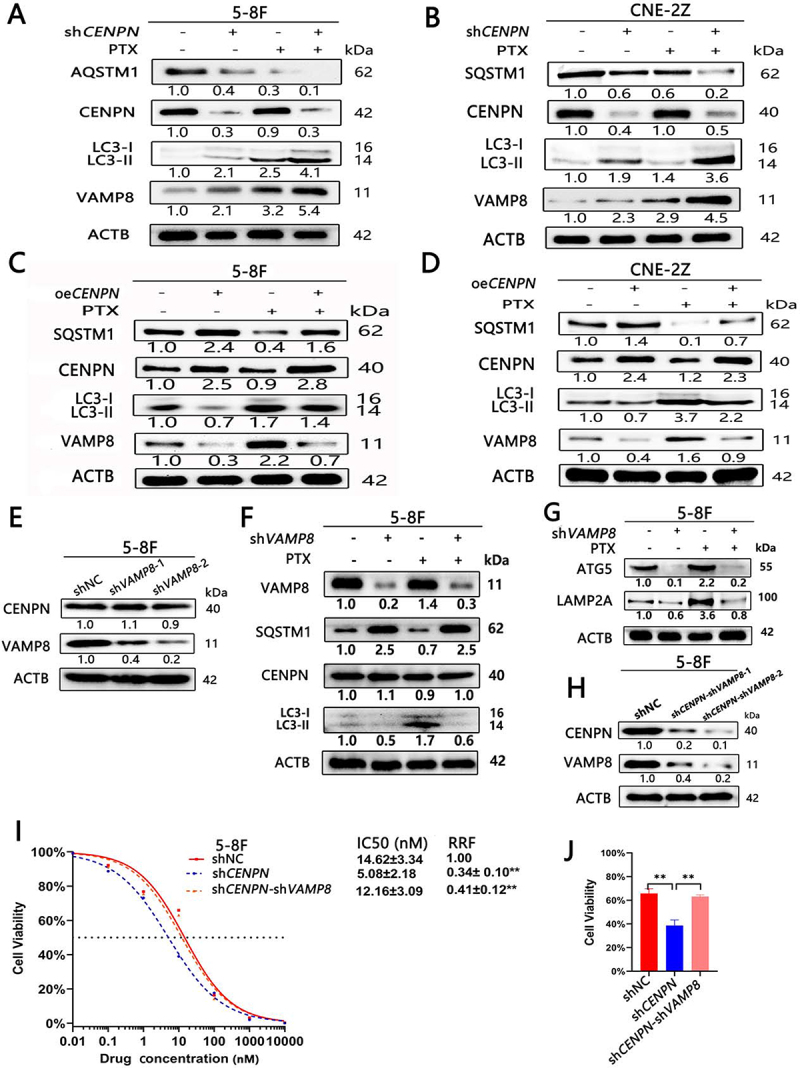
CENPN affects autophagy through VAMP8 and then leads to PTX resistance in NPC cells. (A, B) Western blot analysis showed that PTX (10 nM) significantly affected the levels of VAMP8 and autophagy in NPC cells after *CENPN* knockdown. (C, D) Western blot analysis showed that PTX (5 nM) significantly affected the levels of VAMP8 and autophagy in NPC cells after *CENPN* overexpression. (E) 5-8F shVAMP8 cells were successfully generated. (F, G) The changes of autophagy and PTX (5 nM) sensitivity of NPC cells after knockdown of *VAMP8* were detected by western blot. (H) 5-8F sh*CENPN*-sh*VAMP8* cells were successfully generated. (I) The cytotoxicity assay showed that the PTX sensitivity of 5-8F sh*CENPN*-sh*VAMP8* cells was significantly lower than that of 5-8F sh*CENPN* cells. (J) The CCK8 assay showed that the viability of 5-8F sh*CENPN*-sh*VAMP8* cells was significantly increased compared to that of 5-8F sh*CENPN* cells. The concentration of PTX was 10 nM. Data are presented as mean ± SD. *, P<0.05. **, P<0.01.

In 5-8F and CNE-2Z cells, both VAMP8 expression and autophagy were significantly increased in the shNC+PTX group compared with the shNC group (LC3-II:LC3-I was increased, and SQSTM1 was decreased). Both VAMP8 expression and autophagy were significantly increased in the sh*CENPN* group compared with the shNC group. VAMP8 expression and autophagy were significantly increased in the sh*CENPN*+PTX group compared with the sh*CENPN* group (Figure 6A-B).

In 5-8F and CNE-2Z cells, both VAMP8 expression and autophagy were significantly increased in the oeVec+PTX group compared with the oeVec group (LC3-II:LC3-I was increased, and SQSTM1 was decreased). VAMP8 expression and autophagy were reduced in the oe*CENPN* group compared with the oeVec group. VAMP8 expression and autophagy were significantly decreased in the oe*CENPN*+PTX group compared with the oeVec+PTX group (Figure 6C-D).

Compared with that in the shNC group, VAMP8 protein expression in the small hairpin RNA expression vector targeting human *VAMP8* gene (sh*VAMP8*) group was significantly decreased, but CENPN protein expression was not significantly decreased, indicating that knockdown of *VAMP8* alone does not affect the expression of CENPN (Figure 6E). Since PTX promoted the expression of VAMP8 and the level of autophagy in 5-8F and CNE-2Z (Figure 6A–-6D), it suggested that PTX could promote VAMP8 expression in NPC. Therefore, we examined the effect of knockdown of VAMP8 on autophagy and PTX sensitivity in NPC. Compared with shNC group, autophagy in sh*VAMP8* group was decreased (LC3-II:LC3-I, LAMP2A and ATG5 were decreased, SQSTM1 was increased). Compared with shNC+PTX group, autophagy of sh*VAMP8*+PTX group was decreased. Compared with sh*VAMP8* group, autophagy of sh*VAMP8*+PTX group was not significantly changed (Figure 6F-G). These results indicate that PTX can improve the autophagy by promoting VAMP8 expression in NPC.

Next, lentiviral transduction was used to generate two lines of 5-8F cells with sequential knockdown of *CENPN* and *VAMP8* (sh*CENPN*-sh*VAMP8*-1 and sh*CENPN*-sh*VAMP8*-2). Western blot analysis confirmed that the CENPN and VAMP8 expression levels were significantly decreased in both cell lines (Figure 6H). sh*CENPN*-sh*VAMP8*-2 (referred to as sh*CENPN*-sh*VAMP8*), with the best knockdown effect, was selected for the following experiments. The results of the PTX cytotoxicity assay and IC50 assay showed that the sh*CENPN*-sh*VAMP8* transduction reversed the sensitivity of sh*CENPN* cells to PTX, and the IC50 and RFF did not differ significantly between the sh*CENPN*-sh*VAMP8* group and shNC group and were higher in both of these groups than in the sh*CENPN* group (Figure 6I). When the concentration of PTX was 10 nM, cell viability in the sh*CENPN* group (39.69±3.98%) was significantly lower than that in the shNC group (65.80±3.98%) and sh*CENPN*-sh*VAMP8* group (63.09±3.01%) (Figure 6J).

### *Sequential knockdown of* CENPN *and* VAMP8 *can reverse the enhancement of autophagy induced by knockdown of* CENPN *in NPC cells*

**Figure 7.**
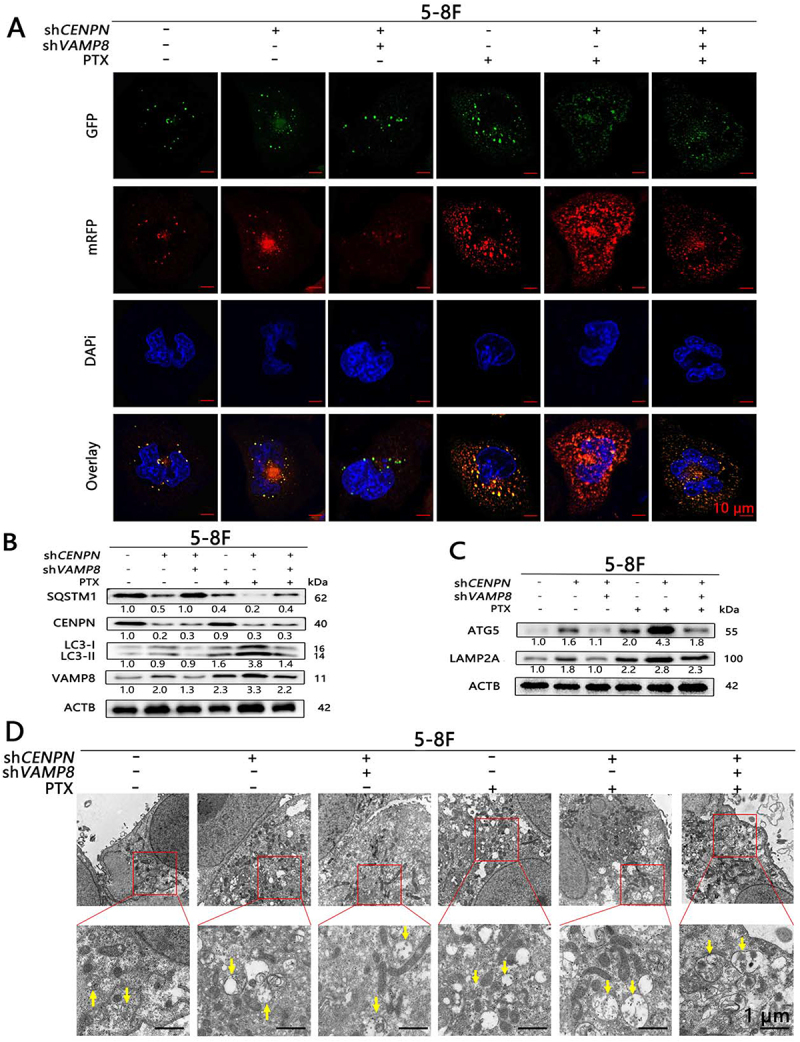
Sequential inhibition of *CENPN* and *VAMP8* expression reverses the increase in autophagy after inhibition of *CENPN* expression alone. (**A**) The tandem mRFP-GFP-LC3 reporter assay showed that autophagic flux in sh*CENPN*-sh*VAMP8* cells was significantly reduced compared with that in sh*CENPN* cells. (**B, C**) Western blot analysis showed that the levels of autophagy in sh*CENPN*-sh*VAMP8* cells were significantly altered compared with those in sh*CENPN* cells. (**D**) TEM showed that the number of autophagosomes in sh*CENPN*-sh*VAMP8* cells was significantly reduced compared with that in sh*CENPN* cells (3000×, 8000×). The yellow arrows indicate typical autophagosomes. Data are presented as mean ± SD.*, P < 0.05. **, P < 0.01.

The tandem mRFP-GFP-LC3 reporter assay and TEM showed that before PTX treatment, the numbers of autophagosomes and autolysosomes in the shNC group and the sh*CENPN*-sh*VAMP8* group were not significantly different and were significantly lower than those in the sh*CENPN* group. After treatment with 10 nM PTX, the numbers of autophagosomes and autolysosomes in both the shNC group and sh*CENPN*-sh*VAMP8* group were significantly increased but were significantly lower than those in the sh*CENPN* group (Figure 7A,D, Fig. S8A-B). WB analysis showed that there were no significant differences in the levels of autophagy between the shNC group and the sh*CENPN*-sh*VAMP8* group and that the levels of autophagy in both of these groups were significantly decreased compared with that in the sh*CENPN* group (LC3-II:LC3-I, LAMP2A, and ATG5 were decreased, while SQSTM1 was increased). After treatment with 10 nM PTX, the levels of autophagy in both the shNC group and sh*CENPN*-sh*VAMP8* group was increased but was significantly lower than that in the sh*CENPN* group (Figure 7B,C). These results confirmed that knockdown of both *CENPN* and *VAMP8* in 5-8F cells can reverse the increase of autophagy caused by knockdown of *CENPN*, thereby reducing the PTX-sensitizing effect of sh*CENPN*.

Moreover, there were no significant differences in cell viability, clonal proliferation ability, or cell cycle progression between the sh*CENPN*-sh*VAMP8* group and the shNC group, and all were significantly increased in both of these groups compared with the sh*CENPN* group; in addition, there was no significant difference in PTX sensitivity, which was decreased in both of these groups compared with the sh*CENPN* group (Fig. S8C-K).

### *Knockdown of* CENPN *reduces PTX resistance in NPC xenografts by activating VAMP8 to promote autophagy*

**Figure 8.**
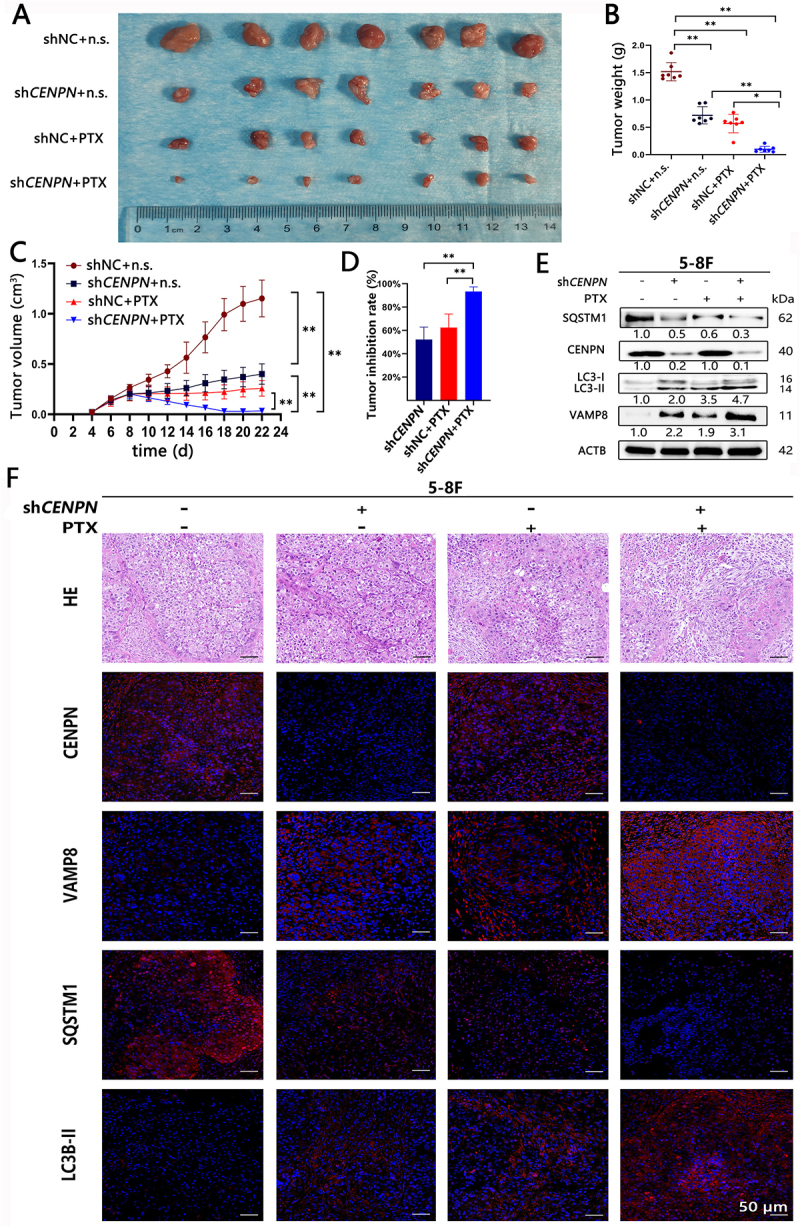
Knockdown of *CENPN* significantly increases the sensitivity of NPC xenografts to PTX. (A) NPC xenografts in each group were harvested at the end of the experiment. (B) Comparison of NPC xenograft weights in each group at the end of the experiment. (C) Growth curves of NPC xenografts in each group. (D) The tumor inhibition rate was significantly increased after PTX treatment in the *CENPN* knockdown group. (E) WB analysis showed the levels of autophagy in NPC xenografts in each group. (F) HE and immunofluorescence staining were used to evaluate the levels of autophagy in NPC xenografts in each group (200×). n.s., normal saline. Data are presented as mean ± SD. **, P<0.01.

The tumor growth rate and tumor volume were significantly reduced, and the sensitivity to PTX was significantly increased in the sh*CENPN* group compared with the shNC group (Figure 8A-C). The TIR in the sh*CENPN*+PTX group (93.34±3.68%) was significantly higher than that in the shNC+PTX group (62.43±10.75%) and the sh*CENPN* group (52.20±9.92%), all of which were higher than that in the shNC group (Figure 8D).

Compared with those in the shNC group, the levels of autophagy and VAMP8 in the sh*CENPN* group were significantly increased, and the levels of autophagy and VAMP8 were further enhanced after PTX treatment (Figure 8E-F, Fig. S9). These results confirmed that knockdown of *CENPN* can increase the sensitivity of NPC xenografts to PTX by increasing VAMP8 expression and enhancing autophagy.

### *Knockdown of* CENPN *enhances the sensitivity of NPC to PTX by increasing CREB/VAMP8 expression to promote autophagy*

**Figure 9.**
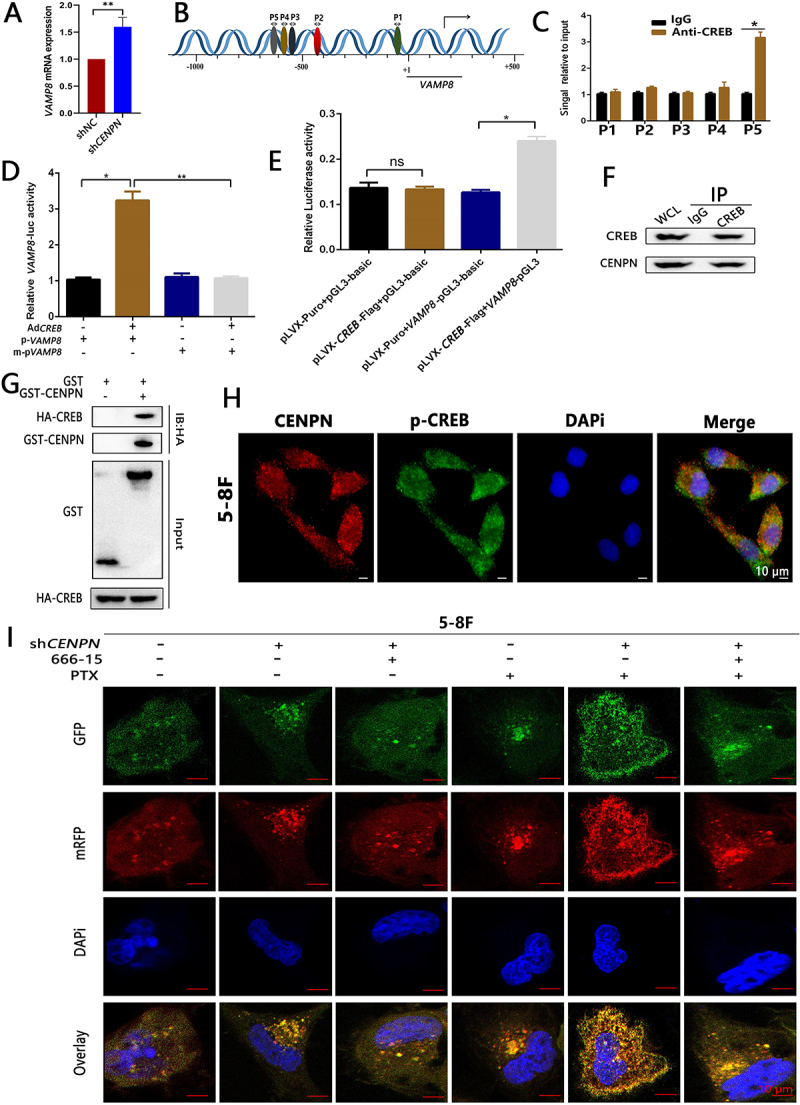
Knockdown of *CENPN* activates CREB/VAMP8 to enhance autophagy and thus increase the PTX sensitivity of NPCs. (A) qRT‒PCR showed that 5-8F sh*CENPN* cells exhibited higher *VAMP8* mRNA levels than control cells. (B) Bioinformatics analysis predicted that the promoter sequence of the *VAMP8* gene may bind to CREB. (C) ChIP-PCR showed that CREB could bind to the *VAMP8* promoter sequence. (D-E) A luciferase reporter assay was used to determine the effect of the CREB binding site on the promoter region of *VAMP8*. (F) Co-IP assay showed a protein‒protein interaction between CENPN and CREB in 5-8F cells. (G) GST affinity-isolation assays showed a direct protein‒protein interaction between CENPN and CREB in 5-8F cells. (H) A cellular immunofluorescence colocalization assay showed that CENPN and p-CREB proteins were colocalized in 5-8F cells. It was representative of 48 cells. (I) Tandem mRFP-GFP-LC3 reporter showed that 666-15 inhibited the increase in autophagy and PTX sensitivity in 5-8F sh*CENPN* cells. The concentrations of 666-15 were 1.2 μΜ. The concentrations of PTX were 10 nM. Data are presented as mean ± SD.*, P < 0.05. **, P < 0.01. ns, no significance.

qRT‒PCR showed that the *VAMP8* mRNA level increased after knockdown of *CENPN* (Figure 9A), confirming that CENPN inhibits *VAMP8* transcription. We found through bioinformatics that five promoter sites of the *VAMP8* gene (P1-P5) may bind to the transcription factor CREB and thus speculated that CREB may be a transcription factor for *VAMP8* (Figure 9B). Chromatin immunoprecipitation (ChIP)-PCR subsequently confirmed that CREB could bind to the P5 site (-601 to ≈-697) in the *VAMP8* promoter in 5-8F cells (Figure 9C). A luciferase reporter assay showed that the relative *VAMP8* luciferase activity in the Ad*CREB*+p-*VAMP8* group was significantly higher than that in the p-*VAMP8* group, m-p*VAMP8* group and Ad*CREB*+m-p*VAMP8* group (Figure 9D). The relative luciferase activity of pLVX-*CREB*-Flag+*VAMP8*-pGL3 cotransfected cells was significantly higher than that of pLVX-Puro+*VAMP8*-pGL3-Basic cotransfected cells (Figure 9E). These results confirmed that CREB is a *VAMP8* transcription factor that binds to specific sites in the *VAMP8* promoter sequence.

We next explored whether CENPN regulates CREB expression. A co-IP assay showed a protein-protein interaction between CENPN and CREB in 5-8F cells (Figure 9F). GST affinity-isolation assays revealed a direct protein‒protein interaction between CENPN and CREB in 5-8F cells (Figure 9G). Cellular immunofluorescence colocalization assays showed that CENPN and p-CREB were colocalized in 5-8F cells (r=0.88) (Figure 9H).

A tandem mRFP-GFP-LC3 reporter assay showed that the CREB phosphorylation inhibitor 666-15 significantly attenuated the increase in the number of autophagosomes and autolysosomes in NPC cells caused by knockdown of *CENPN*. 666-15 also reduced the PTX sensitivity of NPC cells (Figure 9I, Fig. S10A-B). These results confirmed that the enhancement of autophagy in NPC caused by knockdown of *CENPN* was closely related to the phosphorylation of CREB.

### *The specific binding of CENPN to p-CREB in the cytoplasm leads to a reduction in CENPN translocation into the nucleus, which leads to a reduction in* VAMP8 *transcription and autophagy.*

**Figure 10.**
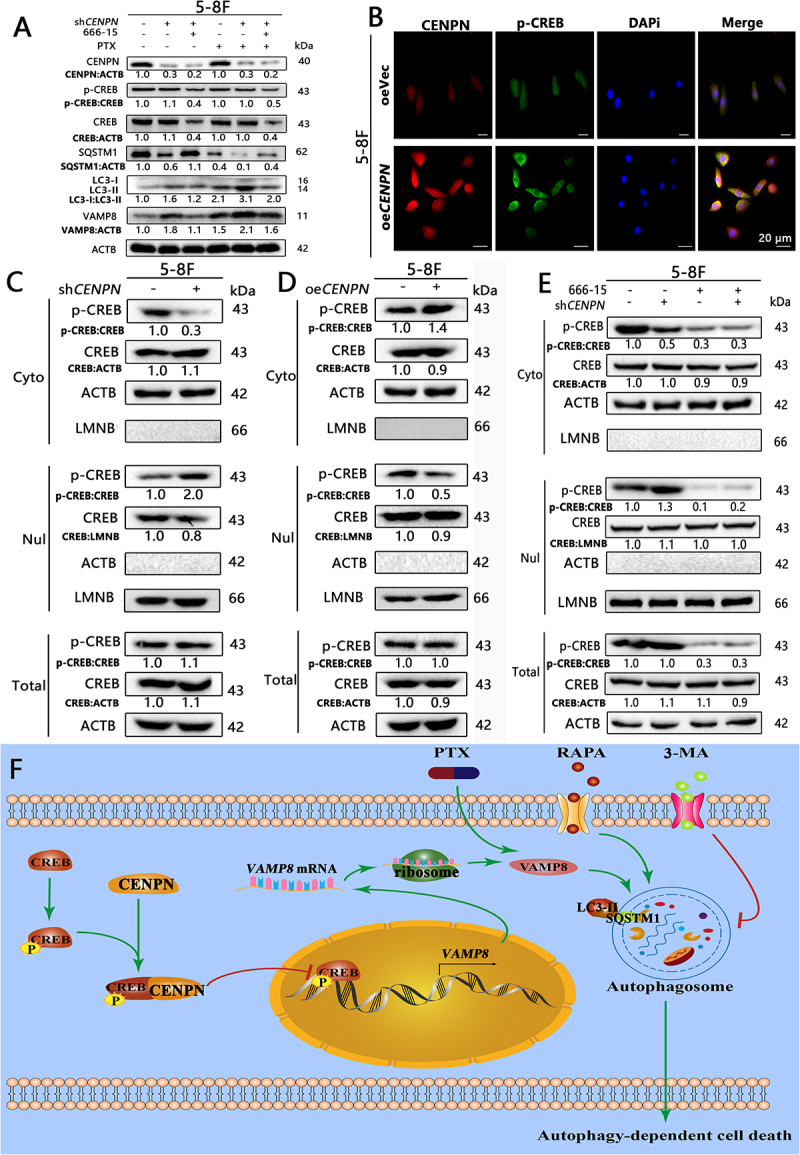
CENPN binding to p-CREB inhibits *VAMP8* transcription and thus suppresses autophagy in NPCs. (A) Western blot analysis showed the effect of 666-15 on p-CREB: CREB, VAMP8, LC3-II:LC3-I and SQSTM1 in 5-8F sh*CENPN* cells. (B) A cellular immunofluorescence colocalization assay was used to analyze the colocalization of CENPN and p-CREB in 5-8F oe*CENPN* cells. (C) Western blot analysis showed the differential expression of p-CREB and CREB in the cytoplasm and nucleus in 5-8F sh*CENPN* cells. (D) Western blot analysis showed the differential expression of p-CREB and CREB in the cytoplasm and nucleus in 5-8F oe*CENPN* cells. (E) Western blot analysis showed the effect of 666-15 on p-CREB: CREB expression in the cytoplasm and nucleus in 5-8F sh*CENPN* cells. (F) Mechanistic diagram of PTX resistance induced by CENPN in NPC. The concentrations of 666-15 were 1.2 μΜ. The concentrations of PTX were 10 nM. *, P < 0.05. **, P < 0.01.

Compared with that in the shNC group, the levels of VAMP8 and autophagy in the sh*CENPN* group were increased, and 666-15 significantly blocked the increase in the levels of VAMP8 and autophagy in the sh*CENPN* group. 666-15 significantly attenuated the PTX-induced increase in VAMP8 and autophagy in the sh*CENPN* group (Figure 10A). 666-15 significantly inhibited *CENPN* knockdown-induced decline of cell survival, clonal proliferation, cell cycle and increase of apoptosis in NPC (Fig. S10C-K).

Cellular immunofluorescence colocalization assays showed that p-CREB in the oeVec group mainly accumulated in the cytoplasm, and p-CREB in the oe*CENPN* group mainly accumulated outside the nucleus (Figure 10B). Compared with that in the shNC group, the p-CREB: CREB ratio in the cytoplasm in the sh*CENPN* group was significantly decreased, the p-CREB: CREB ratio in the nucleus was significantly increased, and the total p-CREB: CREB ratio had no significant change (Figure 10C). Compared with that in the oeVec group, the p-CREB: CREB ratio was significantly increased in the cytoplasm and decreased in the nucleus in the oe*CENPN* group, and the total p-CREB: CREB ratio showed no significant change (Figure 10D). 666-15 significantly decreased the p-CREB and CREB levels in the cytoplasm and nucleus of the shNC group and sh*CENPN* group (Figure 10E). Thus, we confirmed that CENPN inhibits p-CREB entry into the nucleus by direct protein‒protein binding, thereby reducing the transcriptional activity of *VAMP8*, leading to PTX resistance and a poor prognosis in NPC patients (Figure 10F).

## Discussion

Studies have shown that abnormal expression of CENPN is related to the pathogenesis of NPC, breast cancer, and oral squamous cell carcinoma [24,32–34]. However, the relationship between CENPN and tumor chemotherapeutic resistance has not yet been reported. This study showed that the expression of CENPN in NPC patients was positively correlated with PTX resistance and poor prognosis. This study found that knockdown of *CENPN* significantly increased the sensitivity of NPC cells to PTX, while overexpression of *CENPN* significantly decreased the sensitivity to PTX. Thus, we confirmed that CENPN expression can result in PTX resistance and poor prognosis in NPC. LC3, SQSTM1 and ATG5 in cancer tissues are closely related to cancer aggressiveness and prognosis, and are often used as independent prognostic markers for cancer [35-37]. Our study showed that, compared with those in PTX-resistant NPC patients, the expression levels of LC3-II and ATG5 were significantly higher while the expression levels of SQSTM1 were significantly lower in PTX-sensitive NPC patients. Thus, we confirmed that PTX resistance and poor prognosis in NPC patients were associated with inhibition of autophagy by CENPN.

The clinical tissue analysis in this study confirmed that the CENPN expression level exhibited a negative correlation with the level of autophagy in NPC cells. Overexpression of *CENPN* resulted in attenuated autophagy and reduced sensitivity to PTX, while RAPA reversed the decreased autophagy level and paclitaxel sensitivity caused by overexpression of *CENPN* in NPC cells. Knockdown of *CENPN* resulted in enhanced autophagy and increased sensitivity to PTX, while both 3-MA (autophagy inhibitor in the initial stage) and CQ (autophagy inhibitor in the final stage) could inhibit the activation of autophagic flux and the increase sensitivity to paclitaxel induced by *CENPN* knockdown.

Thus, we confirmed that PTX resistance and poor prognosis in NPC are closely related to CENPN-mediated inhibition of autophagy. Kuang et al. found that BST2 can induce cisplatin resistance in NPC and that cisplatin does not affect the expression of BST2, thus suggesting that BST2 may be an intrinsic factor rather than an acquired factor that mediates cisplatin resistance [38]. Our study showed that PTX does not affect the expression of CENPN, which also showed that CENPN-mediated PTX resistance is an intrinsic characteristic of NPC cells that is not affected by the external environment, such as exposure to chemotherapeutic drugs.

RNA-seq can identify differentially expressed genes and differentially activated signaling pathways during tumor formation as well as target genes related to tumor therapeutic resistance [39,40]. We used RNA-seq to explore the types of genes downregulated after inhibition of *CENPN* expression and investigate their effects on NPC cell function [24]. We have analyzed the types of genes up-regulated after inhibiting *CENPN* expression and their effects on NPC cell function in this study. We found that *VAMP8* expression was significantly increased after inhibition of *CENPN* expression in NPC cells. Through evaluation of the numbers of autophagosomes and autolysosomes and the levels of autophagy, we confirmed that CENPN suppresses autophagy by inhibiting the expression of VAMP8, thereby reducing chemosensitivity to PTX. We also found that PTX enhanced autophagy in NPC cells by increasing VAMP8. Chen et al. found that the phosphorylation-deficient mutant of VAMP8, VAMP8Ala, blocks lysosome-autophagosome fusion and increases temozolomide resistance in HeLa cells by suppressing autophagy [31]. The results of this study were similar to those in that report. Thus, we confirmed that CENPN reduces autophagy by downregulating VAMP8 and thereby suppressing the fusion of autophagosome with lysosome, resulting in PTX resistance and poor prognosis in NPC patients.

Yang et al. showed that sequential knockdown of two genes has a more pronounced effect on tumor cells than simultaneous knockdown of the same two genes [19]. We found that sequential knockdown of *CENPN* and *VAMP8* in NPC cells blocked the enhancement of autophagy and inhibition of cell phenotypes induced by knockdown of *CENPN* as well as the PTX-sensitizing effect of *CENPN* knockdown. Interestingly, there was no significant change in CENPN expression after knockdown of *VAMP8*, confirming that the inhibition of autophagy by CENPN is dependent on downstream VAMP8 expression. qRT-PCR, ChIP and the luciferase reporter assay showed that CENPN can significantly reduce the ability of VAMP8 promoter binding and *VAMP8* transcriptional activity, thereby reducing autophagy. These results confirmed that the specific mechanism by which CENPN regulates PTX resistance in NPC is mediated via CENPN-induced repression of VAMP8 transcription and autophagy suppression.

Zhang et al. showed that 666-15 can significantly inhibit the phosphorylation of CREB [41], and the results of this study are consistent with this finding. In this study, we found that CENPN could specifically bind to p-CREB and inhibit its entry into the nucleus of NPCs, thereby reducing the transcription level of *VAMP8*. The decreased transcription level of *VAMP8* leads to a significant reduction in autophagy, thereby causing chemoresistance in NPC cells. Moreover, our study showed that the level of autophagy in NPC cells was significantly reduced after knockdown of *VAMP8*, and the autophagy induced by PTX was also significantly inhibited. It was confirmed that PTX could directly promote VAMP8 to enhance autophagy, leading to autophagy-dependent cell death in NPC. This study confirmed that there are two main reasons for the enhancement of NPC PTX sensitivity with *CENPN* knockdown. The first is that knockdown of *CENPN* can promote p-CREB translocation into the nucleus and increase the transcription of *VAMP8*, leading to increased autophagy. The second is that PTX itself can promote the protein expression of VAMP8, leading to increased autophagy. The enhancement of autophagy caused by these two pathways leads to autophagy-dependent cell death in NPC cells, thereby achieving the effect of PTX sensitization.

At present, the main treatment approach for NPC is a comprehensive treatment strategy based on chemoradiotherapy. However, recurrence and metastasis are still the main reasons for treatment failure, and delineating mechanisms of chemoresistance to agents such as PTX is the key to improving the clinical outcomes of patients with advanced cancer. This study showed that overexpression of *CENPN* was significantly positively correlated with chemotherapeutic resistance and poor prognosis in NPC patients. Some small molecule inhibitors targeting oncogenes have achieved good curative effects in the clinical treatment of cancer and are expected to have good development prospects in the next decade [42]. Considering the results of this study, the development of small molecule drugs that specifically target CENPN may have good clinical application prospects in the treatment of advanced NPC. In addition, the chemosensitizing effect of CENPN in other tumors or with other chemotherapeutic drugs (such as platinum compounds) also deserves further study. For example, most ovarian cancer patients receive PTX chemotherapy, but only 42% of patients respond to PTX [43], and the relationship between CENPN and PTX resistance in ovarian cancer is also worthy of further study.

In conclusion, this study verified that CENPN suppresses autophagy by inhibiting VAMP8 expression, causing PTX resistance and poor prognosis in NPC. CENPN may be a predictive marker of chemotherapeutic efficacy and a potential target for inducing chemosensitization to agents such as PTX.

## Materials and methods

### TMA

The TMA (Guilin Fanpu Biotechnology, NPC131) contained samples from a total of 131 patients—32 with NPG and 99 with NPC, including 45 NPC patients with 10-year follow-up data. The TMA was immunohistochemically stained and scored according to the intensity of tissue staining (0, 1, 2, and 3 points for unstained, pale yellow, tan, and dark brown staining, respectively) and the percentage of stained cells (1, 2, and 3 points for< 10%, 10%-50%, and >50% stained cells, respectively). The final assessment of the staining extent (0, 1-3, 4-6, and 7-9 points for negative, weak, positive, and strong positive staining, respectively) was then performed by multiplying the staining intensity score by the percentage of stained cells score [44]. Negative and weakly positive samples were classified into the CENPN low expression group, and positive and strongly positive samples were classified into the CENPN overexpression group [24].

### Clinical organization

A retrospective analysis of 35 NPC patients admitted to the Oncology Department of Renmin Hospital of Wuhan University from January 2021 to December 2021 was performed. Inclusion criteria [45]: (i) NPC diagnosed by pathological examination, (ii) no distant metastasis, (iii) treatment with 2-3 cycles of PTX-based neoadjuvant chemotherapy (NAC), (iv) complete MRI data before and after NAC, (v) measurable lesions on nasopharyngeal MRI before NAC, and (v) no prior anticancer therapy. Exclusion criteria: (i) refusal or failure to complete NAC, (ii) metastasis at diagnosis. According to the RECIST 1.1 efficacy evaluation criteria, among the 35 patients included in the study, 16 patients evaluated to have partial remission or complete remission were classified into the chemotherapy-sensitive group, and 19 patients evaluated to have partial progression or stable disease were classified into the chemotherapy-resistant group. This study was approved by the Ethics Committee of Renmin Hospital of Wuhan University [2020K-K221(Y01)], and all patients provided informed consent. Treatment regimens were performed according to the 2019 USA National Comprehensive Cancer Network (NCCN) guidelines.

### RNA-seq

With reference to the literature [24], the Illumina HiSeq sequencing platform (Genergy Biotechnology, Shanghai) was used to perform high-throughput RNA-seq on NPC cell samples with and without knockdown of *CENPN*. The sequence alignment results were screened to identify differentially expressed genes using DESeq2 software, and the screening criteria were |log2FC|≥1 and P<0.05.

### Cell culture and handling

The NPC cell line 5-8F is a highly metastatic human cell line (gifted from Southern Medical University), and the NPC cell line CEN-2Z is a poorly differentiated human cell line (Shanghai Genechem, GCPC0142923). The medium contained 1% penicillin-streptomycin and 10% fetal bovine serum (Gibco, 10270-106), and the incubator environment was 37°C, 5% CO_2_, and saturated humidity. NPC cell lines were stored in liquid nitrogen at the Institute of Otolaryngology and Head and Neck Surgery, Renmin Hospital of Wuhan University.

### Generation of knockdown and overexpression cells

293T cells (gifted from the College of Life Science, Wuhan University) were used for lentiviral packaging; viral supernatants were collected after 48 h; and 24 h after transfection of NPC cells, cells were selected with puromycin (MedChemExpress, HY-B1743A) or G418 (MedChemExpress, HY-K1056) for 14 days prior to expansion.

### CCK-8, IC50 and clonal proliferation assays

These experiments were used to evaluate the PTX resistance and clonal proliferation ability of NPC cells by Cell Counting Kit (CCK)-8 (YEASEN Biotechnology, 40203ES60). With reference to literature [38], the half-maximal inhibitory concentration (IC50) was determined, and the relative resistance factor (RRF) was calculated by dividing the IC50 in the experimental group by the IC50 in the control group.

### Flow cytometry

Cell Cycle Staining Kit and Annexin V-APC/7-AAD Apoptosis Kit (MultiSciences Biotechnology, AP105) were used to evaluate the cell cycle and cell apoptosis respectively. The detailed procedures were carried out with reference to the literature [46]. Cell cycle data were analyzed with ModFit LT software, and apoptosis was analyzed with CytExpert software (2.3.0.84).

### Detection of autophagic flux

The tandem mRFP-GFP-LC3 reporter is often performed to evaluate the activation of autophagic flux in cells. The yellow spots indicating colocalization of red and green fluorescence are autophagosomes; the red spots are autolysosomes, the yellow spots indicate the formation of mature autophagosomes, and a significant increase in the number of red spots indicates the activation of autophagic flux [47,48]. After NPC cells were infected with mRFP-GFP-LC3 double fluorescent autophagy adenovirus (HanBio Technology, 101508AP), they were treated with PTX (MedChemExpress, HY-B0015), 3-MA (Sigma-Aldrich, HY- 19312), CQ (MedChemExpress, 54-05-7), RAPA (MedChemExpress, HY-10219), 666-15 (MedChemExpress, HY-101120). The cells were cultured in the dark throughout this process. The expression of fluorescent proteins was the highest after 72 h, and images were acquired with a confocal microscope system (Carl Zeiss, Germany).

### TEM

NPC cells treated with different agents were prefixed with 2.5% glutaraldehyde after removal of the medium, scraped gently, collected by centrifugation, postfixed with 2.5% glutaraldehyde, treated with 1% osmium tetroxide, dehydrated with gradient alcohol and acetone, embedded in resin (Servicebio, GP2001), and sectioned (thickness: 65 nm). Autophagosomes were detected by transmission electron microscopy (HITACHI HT7700, Tokyo, Japan).

### WB analysis

Specific steps for protein extraction, detection and analysis to evaluate protein expression were conducted with reference to the literature [44]. The antibodies used are anti-CENPN antibody (Novus biologicals, H00055839-PW1), anti-LC3 antibody (Affinity Biosciences, AF5402), anti-VAMP8 antibody (Abcam, EP2629Y), anti-SQSTM1/p62 antibody (Servicebio, GB11531), anti-ATG5 (Cell Signaling Technology, 2630), anti-LAMP2A (Abcam, ab125068), anti-CREB (Proteintech, 12208-1-AP) and anti-p-CREB antibody (Proteintech, 28792-1-AP).

### Immunohistochemistry

Specific steps for detection and scoring were performed with reference to the literature [46]. The expression level of the target protein in clinical tissues and xenografts was evaluated by calculating the mean absorbance value with ImageJ.

### Immunofluorescence

Specific steps for detection were conducted with reference to the literature [46]. The expression level of the target protein was quantitatively assessed by calculating the tissue’s the mean fluorescence intensity (Mean= IntDen: Area) with Image J [49].

### Xenograft experiment

Twenty-eight 4-week-old male BALB/c nude mice (Shulaibao Biotechnology, 401), were housed in the SPF animal room of the Animal Experiment Center of Renmin Hospital of Wuhan University. According to the specific steps described in the literature [46], the mice were randomly divided into 2 groups (14 mice/group). 5-8F cells expressing negative control shRNA (shNC) and 5-8F cells with *CENPN* knockdown (sh*CENPN*) (5×10^6^ cells/150 μl PBS [Servicebio, G0002-15]/mouse) were subcutaneously injected into the right axillary region of the mice, and the growth of xenografts was monitored every 2 days. When the tumor volume reached approximately 200 mm^3^, the mice in each group were randomly divided into 2 subgroups and received intraperitoneal injection of PTX (10 mg/kg) or an equal volume of normal saline (n.s.; 100 μl) every 2 days for 2 weeks. Then, the mice were killed, and the tumors were removed and weighed. The tumor volume (V) was calculated as follows: V=(a×b×b): 2 [24]. The tumor inhibition rate (TIR) was calculated by the equation TIR = (average tumor weight in control group-average tumor weight in experimental group): average tumor weight in control group × 100% [50]. Animal experiments were approved by the Animal Experiment Ethics Committee of Renmin Hospital of Wuhan University [WDRM No. 20200815].

### qRT-PCR

TRIzol (TIANGEN Biochemical technology, G3013) was used to extract total cellular RNA, mRNA was reverse transcribed into cDNA, and qRT-PCR was performed with a SYBR Green qPCR Kit (Takara Biomedical Technology, 3735A). The relative expression of the target mRNA was calculated by the 2^−ΔΔCT^ method, and the primer sequences are shown in **Table S2**.

### Bioinformatic analysis

Sequences and sites of *VAMP8*-related transcription factors were predicted based on the JASPAR database (https://jaspar.genereg.net/) and UCSC Genome Database (http://genome-asia.ucsc.edu/).

### ChIP

A ChIP assay was used to determine whether CREB could bind to the promoter region of VAMP8. The specific steps were performed according to the literature [51], and the amount of enriched DNA between the IP group and IgG group was analyzed with input as a reference. Primer sequences are shown in **Table S3**.

### Luciferase reporter assay

The luciferase reporter plasmid pGL3-ph*VAMP8* was generated by amplifying the *VAMP8* promoter from the human genome (NC_000002.12) with the primers 5- TAGCCCGGGCTCGAGATCTTTTGAGGCAGAGTCTTGCTCTTGT-3 (forward) and 5-GTACCGGAATGCCAAGCTTGGCCCTCAGTTCACTTCCTG-3 (reverse) and ligating into the pGL3-basic vector (Promega, E1751) between the BgIII and HindIII sites. The CREB binding sites in the *VAMP8* promoter were predicted by bioinformatics analysis. The CREB-binding site sequence deletion plasmid pGL3-ph*VAMP8*-del was generated by fusion-PCR as described earlier with the primers 5-AGGACTTTGGGGAGGATCGCCTGAGCCCA-3 and 5-GCGATCCTCCCCAAAGTCCTAGGAGCCATGATG-3. The 5-8F cells were cotransfected with 100 ng of pGL3-ph*VAMP8* or pGL3-ph*VAMP8*-del and 1 ng of pRL-TK-Luc. The cells were harvested and lysed with 100 μL of passive lysis buffer (Promega, E1941). After removing the cell debris by centrifugation at 13,000 g, for 5 min, the supernatant was detected with the Single-Mode SpectraMax Microplate Reader in accordance with the manufacturer’s instructions (Molecular Devices).

### Co-IP assay

A co-IP assay was used to detect whether the proteins CENPN and CREB interact. In accordance with a previous study [24], 5-8F cells were collected and lysed in RIPA lysis buffer containing a protease inhibitor cocktail; the whole-cell lysate (2 mg) was treated with protein G beads (30 μL; abcam, ab174816), and isotype-matched IgG (2 μg; cabcam ab172730) control or the indicated antibodies were added and incubated for 2 h on a shaking table. The immunoprecipitate was collected after centrifugation. Finally, WB detection was performed.

### GST affinity-isolation assay

This assay was used to detect whether CENPN could directly bind to the target protein CREB. GST-CENPN and HA-CREB genes were first cloned and synthesized and then inserted into pGEX-6P-1 (Sigma, GE28-9546-48). After insert ligation, transformation into recipient cells and clonal expression, the protein samples containing 500 μg GST (control group) or GST-CENPN (experimental group) were added into glutathione-agarose resin (Thermo Scientific, 16100) and mixed for 3 h, respectively. Five hundred micrograms of HA-CREB protein were added to the control and experimental groups and then mixed overnight. The two groups of samples were centrifuged, and an appropriate amount of protein loading buffer was added and then incubated at 100°C for 5 min. Finally, WB experiments were performed with anti-GST (abcam, ab111947) and anti-HA (abcam, ab236632) antibodies for GST: GST-CENPN and HA-CREB, respectively.

### Cellular immunofluorescence colocalization assay

Cells were fixed for 30 min, anti-CENPN primary antibodies (1:200) and anti-p-CREB primary antibodies (1:200) were added, and the cells were incubated overnight at 4°C. Slides were incubated with Cy3-labeled secondary antibody (1:400; Proteintech, 28792-1-AP) for 1 h, followed by Alexa Fluor 488-labeled secondary antibody (1:400; AmyJet Scientific, 115-545-003) for 1 h. Subsequently, the nuclei were stained with DAPI in the dark at room temperature for 15 min before the slides were sealed with an anti-fluorescence quenching solution. Cell staining and protein localization were observed, and images were acquired using a confocal microscope (Olympus, Japan) to select a suitable field of view. Images were then analyzed using ImageJ.

### Statistical analysis

Normally distributed data are expressed as the mean ± standard deviation (mean ± SD) values, and data with a skewed distribution are expressed as medians and quartiles. A t test or one-way ANOVA was used to compare normally distributed measurement data between groups, and the Mann‒Whitney U test was used to compare data with a skewed distribution between groups. Categorical data were analyzed with the χ^2^ test or Fisher’s exact test, and correlations were evaluated by regression analysis; survival analysis was performed with the Kaplan‒Meier method. IBM SPSS (version 26.0) software was used for all statistical analyses, and GraphPad Prism 9.2.2 software was used to plot statistical data. Differences with a two-sided p value of <0.05 were considered significant.

## Supplementary Material

Supplemental Material

## Data Availability

We authors declare all data and materials are available on request.
