# Supplementary material for: CENPN suppresses autophagy and increases paclitaxel resistance in nasopharyngeal carcinoma cells by inhibiting the CREB-VAMP8 signaling axis
Source: Autophagy. 2024 Jan 25;20(2):329–48. doi: 10.1080/15548627.2023.2258052 (PMC10813569; doi:10.1080/15548627.2023.2258052)
Supplement: Supplemental Material [file KAUP_A_2258052_SM9606.docx]

**Table S1.** Basic characteristics of 35 nasopharyngeal carcinoma (NPC) patients between the sensitive and resistant groups.

| Characteristics | Total  (*N* = 35) | Sensitive Group (*N* = 16) | Resistant Group (*N* =19) | P Value |
| --- | --- | --- | --- | --- |
| Age [mean (range)] |  | 55(53,64) | 53(47,59) | 0.108 |
| <60 years | 27 | 11 | 16 | 0.424 |
| ≥60 years | 8 | 5 | 3 |  |
| Gender |  |  |  |  |
| Female | 8 | 5 | 3 | 0.424 |
| Male | 27 | 11 | 16 |  |
| T stage |  |  |  |  |
| T1–2 | 20 | 10 | 10 | 0.557 |
| T3–4 | 15 | 6 | 9 |  |
| N stage |  |  |  |  |
| N0–2 | 29 | 13 | 16 | 8.17 |
| N3 | 6 | 3 | 3 |  |
| Clinical stage |  |  |  |  |
| III | 25 | 12 | 13 | 0.957 |
| IV | 10 | 4 | 6 |  |

**Table S2.** List of primers used for qRT-PCR analysis.

| Genes | Primer | Primer sequence (5’→3’) |
| --- | --- | --- |
| *VAMP8* | Forward primer | TATGACCCAGAATGTGGAGCGG |
| *VAMP8* | Reverse primer | ATTTCCGAGCCACCTTCTGCGA |
| *ACTB* | Forward primer | CACCCAGCACAATGAAGATCAAGAT |
| *ACTB* | Reverse primer | CCAGTTTTTAAATCCTGAGTCAAGC |

**Table S3.** List of primers used for ChIP analysis.

| Promoter site | Primer | Primer sequence (5’–3’) |
| --- | --- | --- |
| P1 | Forward primer | gaggcagagtcttgctcttg |
|  | Reverse primer | gtcaggagttaaagaccagcc |
| P2 | Forward primer | cagggattttagtgctttctgc |
|  | Reverse primer | gtcctaggagccatgatgcc |
| P2 | Forward primer | cagggattttagtgctttctgc |
|  | Reverse primer | gtcctaggagccatgatgcc |
| P4 | Forward primer | caagacattacaaggagatggct |
|  | Reverse primer | gaaagatcaagcagccctcc |
| P5 | Forward primer | cccggcctctttttgcttc |
|  | Reverse primer | ctggataacacttgttcccagg |

**
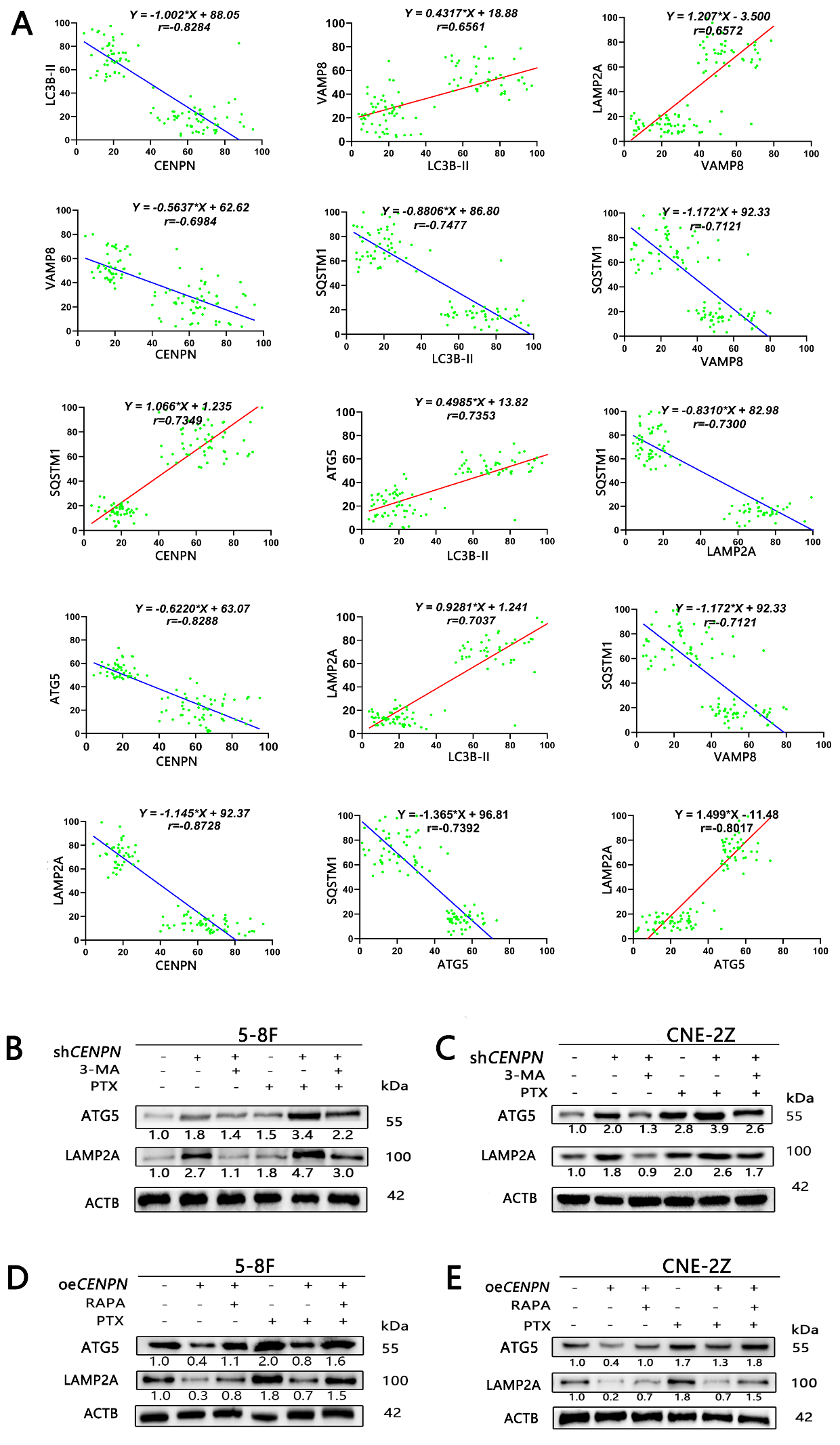
**

**Figure S1.** CENPN expression affects autophagy and PTX resistance in NPC. (**A**) Correlation analysis of CENPN, VAMP8, LC3B-II, SQSTM1, ATG5 and LAMP2A protein levels in NPC tissues. (**B**, **C**) WB analysis showed the effect of PTX (10 nM) on the levels of ATG5 and LAMP2A in NPC cells after knockdown of *CENPN*. (**D**, **E**) WB analysis showed the effect of PTX (5 nM) on the levels of ATG5 and LAMP2A in NPC cells after overexpression of *CENPN*. Data are presented as mean ± SD.*, P<0.05. **, P<0.01.

**
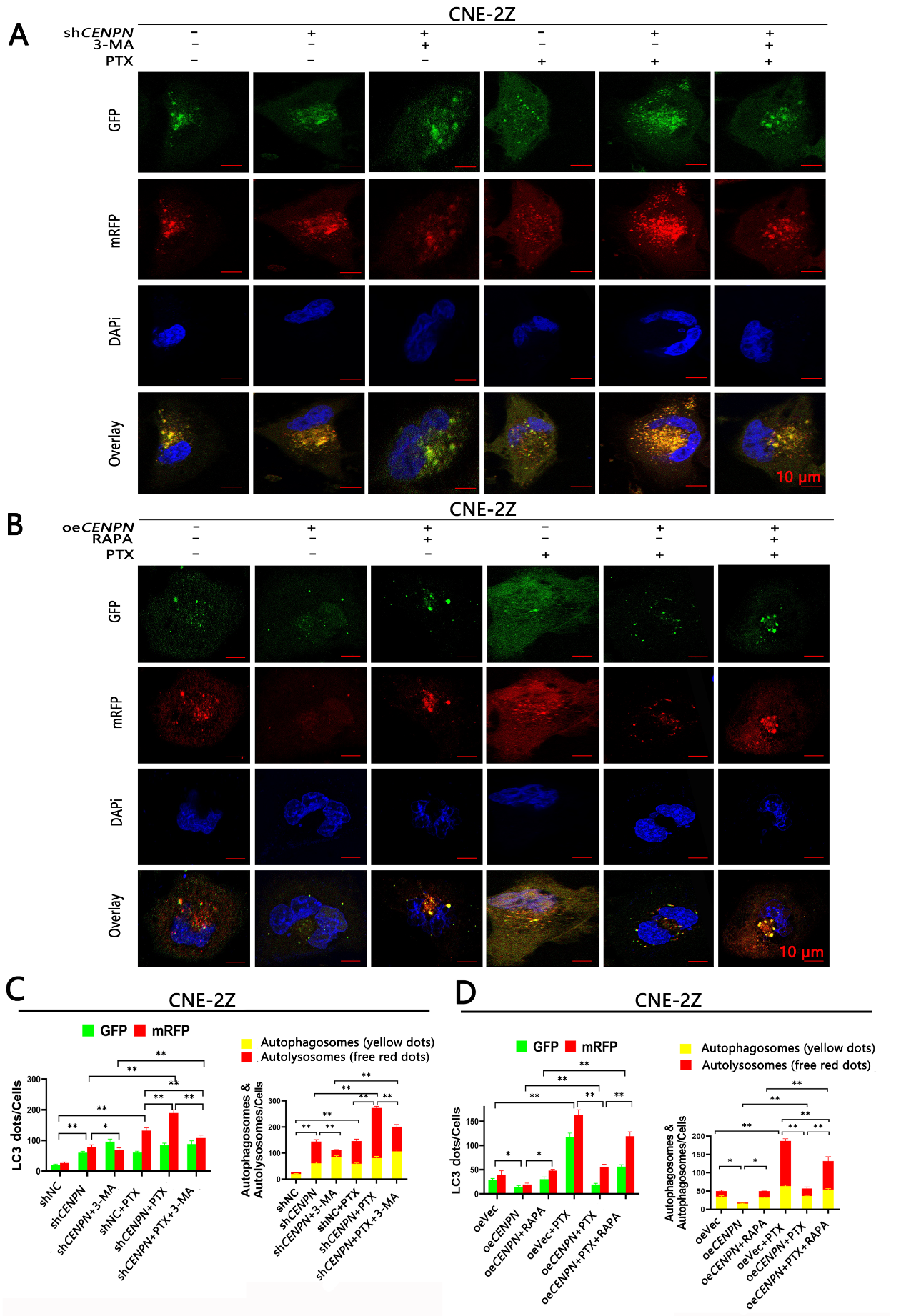
**

**Figure S2**. CENPN expression affects PTX-induced autophagic flux in CNE-2Z cells. (**A**, **C**) The tandem mRFP-GFP-LC3 reporter assay showed that autophagic flux in the sh*CENPN*+PTX group was increased significantly compared with that in the shNC+PTX group. (**B**, **D**) The tandem mRFP-GFP-LC3 reporter assay showed that autophagic flux in the oe*CENPN*+PTX group was decreased significantly compared with that in the oeVec+PTX group. The concentrations of PTX were 10 nM (A, C) and 5 nM (B, D). Data are presented as mean ± SD. *, P<0.05. **, P<0.01.


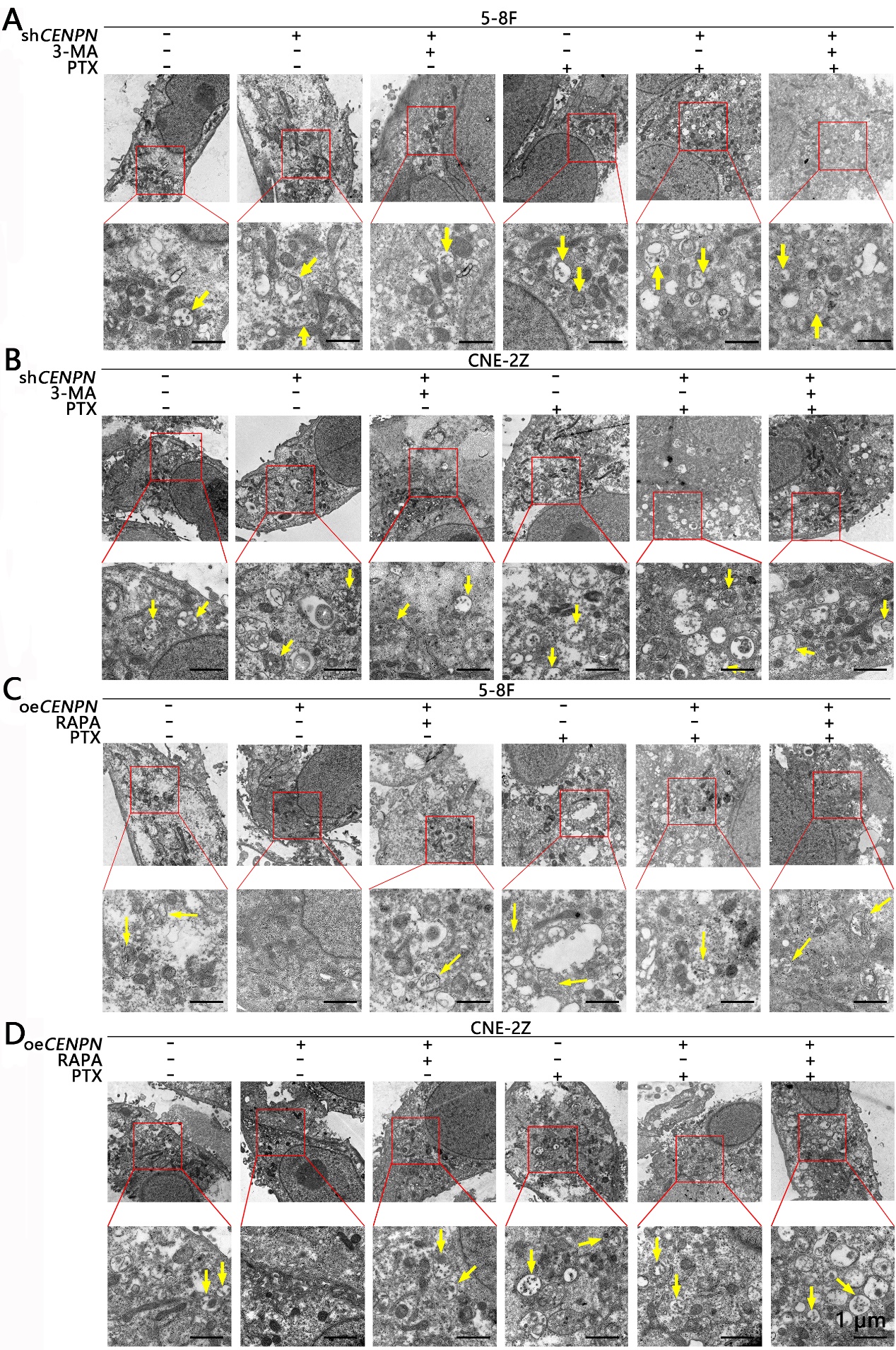


**Figure S3.** CENPN expression affects the number of autophagosomes in NPC cells induced by PTX. (**A**, **B**) TEM showed that the number of autophagosomes in both sh*CENPN* cell lines was significantly higher than that in the control cell line (3000×, 8000×). (**C**, **D**) TEM showed that the number of autophagosomes in both oe*CENPN* cell lines was significantly lower than that in the control cell line (3000×, 8000×). The yellow arrows indicate typical autophagosomes. The concentrations of PTX were 10 nM (A, B) and 5 nM (C, D). *, P<0.05. **, P<0.01.


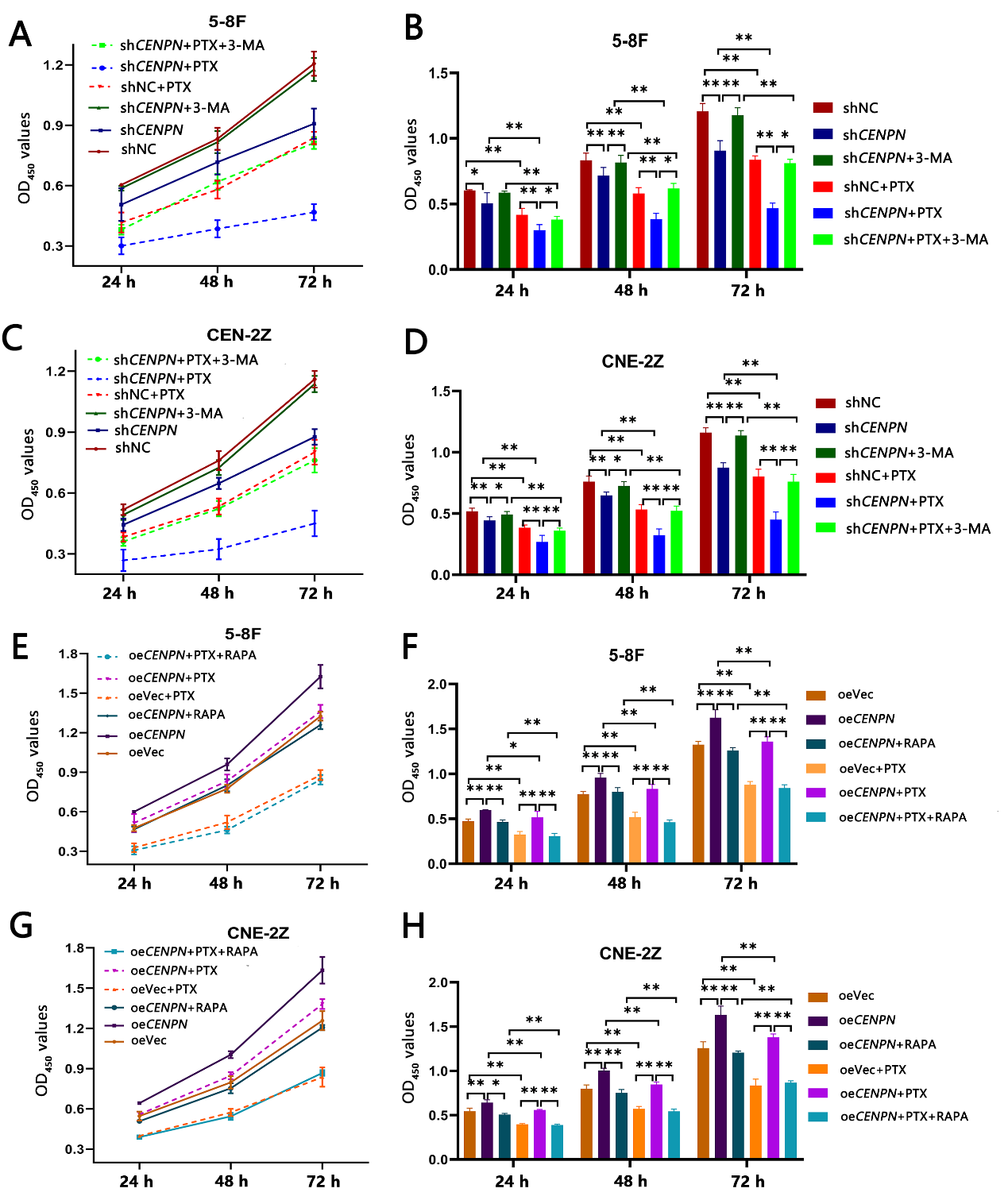


**Figure S4.** CENPN expression increases the viability of NPC cells and reduces their sensitivity to PTX. (**A-D**) The CCK8 assay showed that the viability of sh*CENPN* cells was significantly decreased compared with that of shNC cells, and sh*CENPN* cells were more sensitive to PTX (10 nM). (**E-F**) The CCK8 showed that the viability of oe*CENPN* cells was increased significantly compared with that of oeVec cells, and oe*CENPN* cells were more resistant to PTX (5 nM). Data are presented as mean ± SD. *, P<0.05. **, P<0.01.


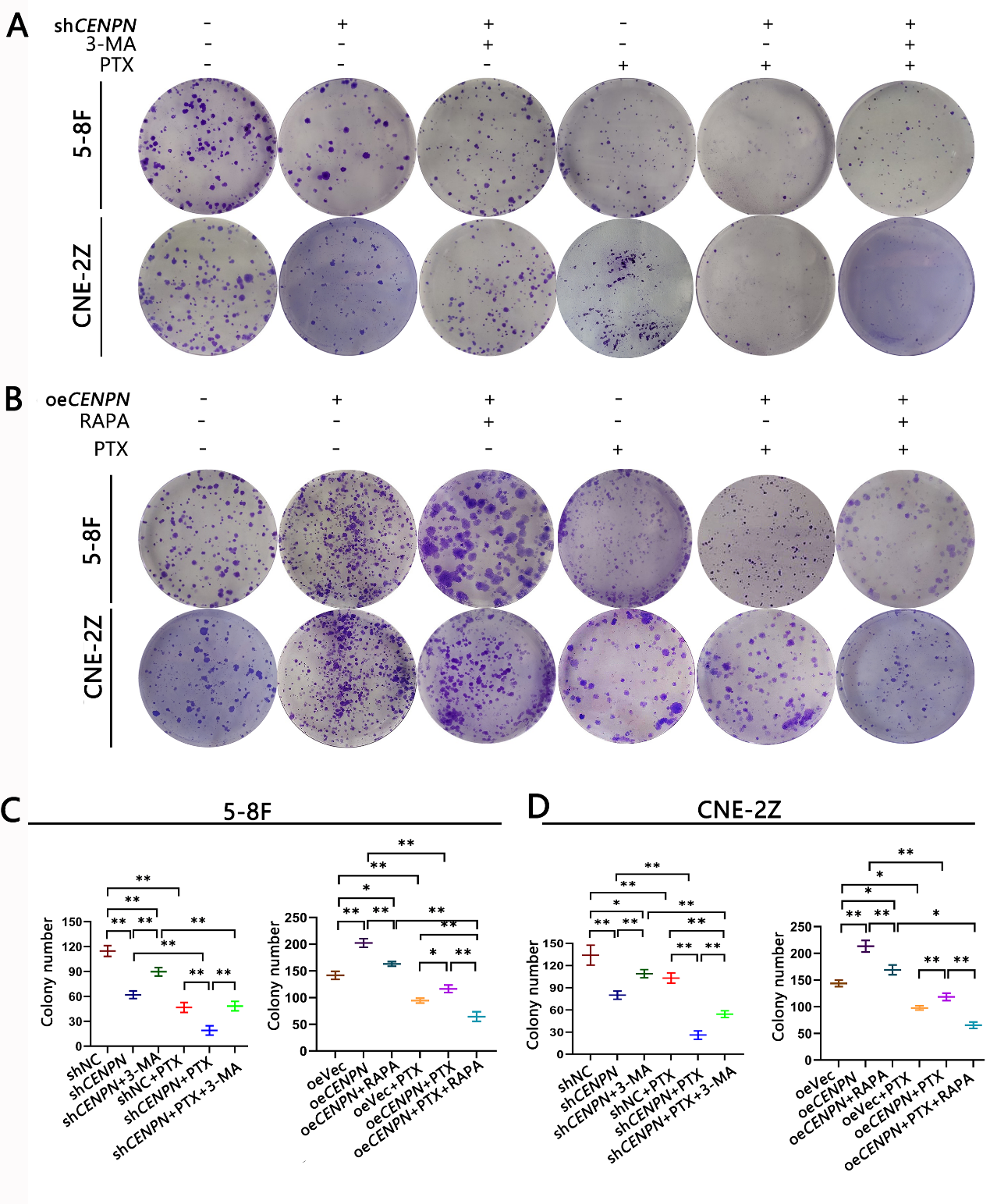


**Figure S5.** CENPN expression promotes the proliferation of NPC cells and reduces their sensitivity to PTX. (**A**, **C**) The clonal proliferation assay showed that the proliferation ability of sh*CENPN* cells was significantly decreased compared with that of shNC cells, and sh*CENPN* cells were more sensitive to PTX (10 nM). (**B**, **D**) The clonal proliferation assay showed that the proliferation ability of oe*CENPN* cells was significantly increased compared with that of oeVec cells, and oe*CENPN* cells were more resistant to PTX (5 nM). Data are presented as mean ± SD. *, P<0.05. **, P<0.01.


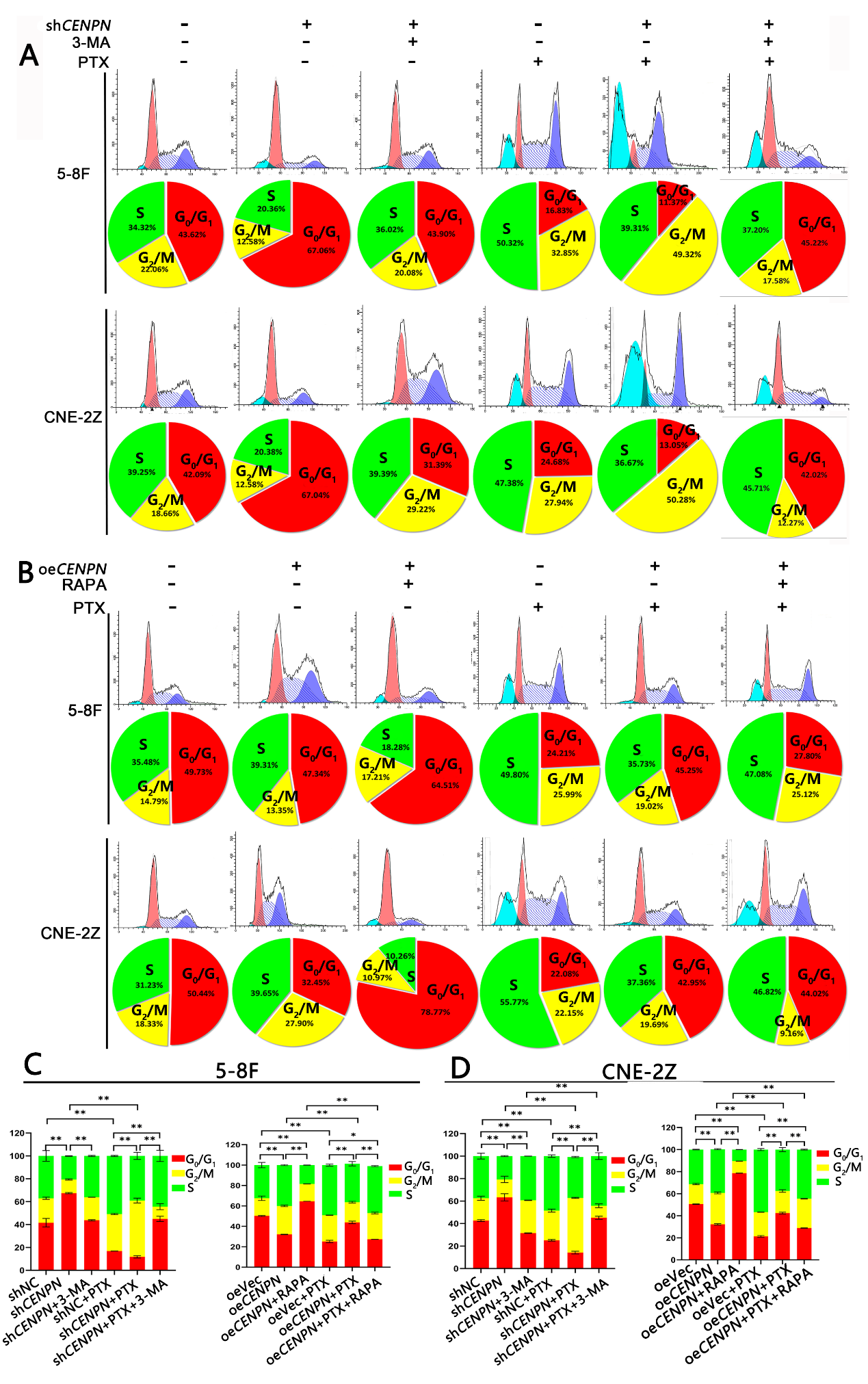


**Figure S6.** CENPN expression promotes cell cycle progression in NPC cells and reduces their sensitivity to PTX. (**A**, **C**) Flow cytometry showed that the percentage of cell cycle arrest in sh*CENPN* cells was significantly increased compared with that in shNC cells, and sh*CENPN* cells were more sensitive to PTX (10 nM). (**B**, **D**) Flow cytometry showed that the percentage of cell cycle arrest in oe*CENPN* cells was decreased significantly compared with that in the shNC group, and oe*CENPN* cells were more resistant to PTX (5 nM). Data are presented as mean ± SD. *, P<0.05. **, P<0.01.


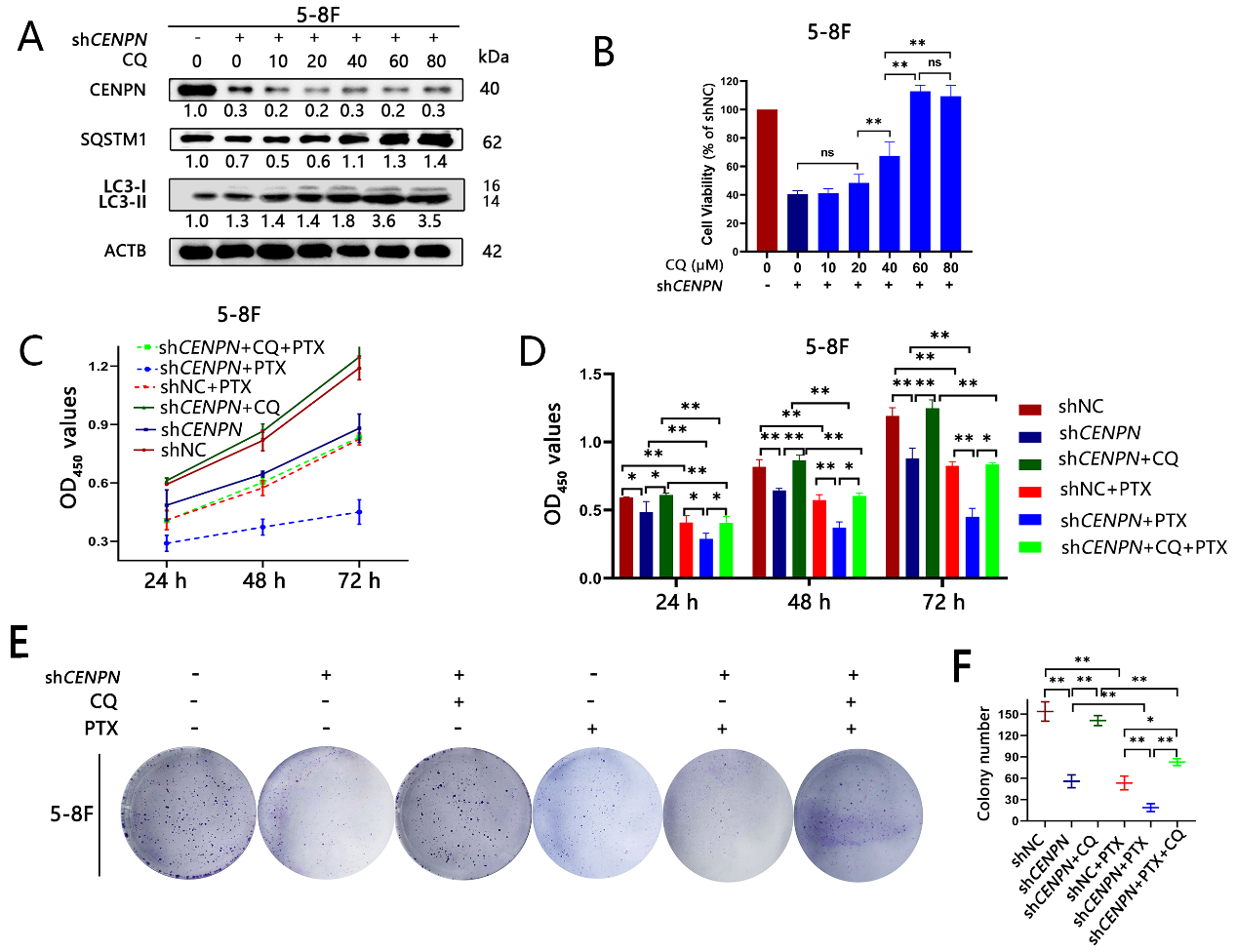


**Figure S7.** Chloroquine [affected](javascript:void(0)) the autophagiy and phenotype in sh*CENPN* cells. (**A**) WB showed that different concentrations of chloroquine (CQ) affected the autophagy in sh*CENPN* cells. (**B**) Cytotoxicity test showed that different concentrations of chloroquine affected the cell viability in sh*CENPN* cells. (**C**, **D**) The CCK8 assay showed that CQ affected the OD values in sh*CENPN* cells. (**E**, **F**) The clone formation assay showed that CQ affected the proliferation ability in sh*CENPN* cells. The concentrations of CQ were 40 μM (C-F). Data are presented as mean ± SD. ns, no significance. *, P<0.05. **, P<0.01.


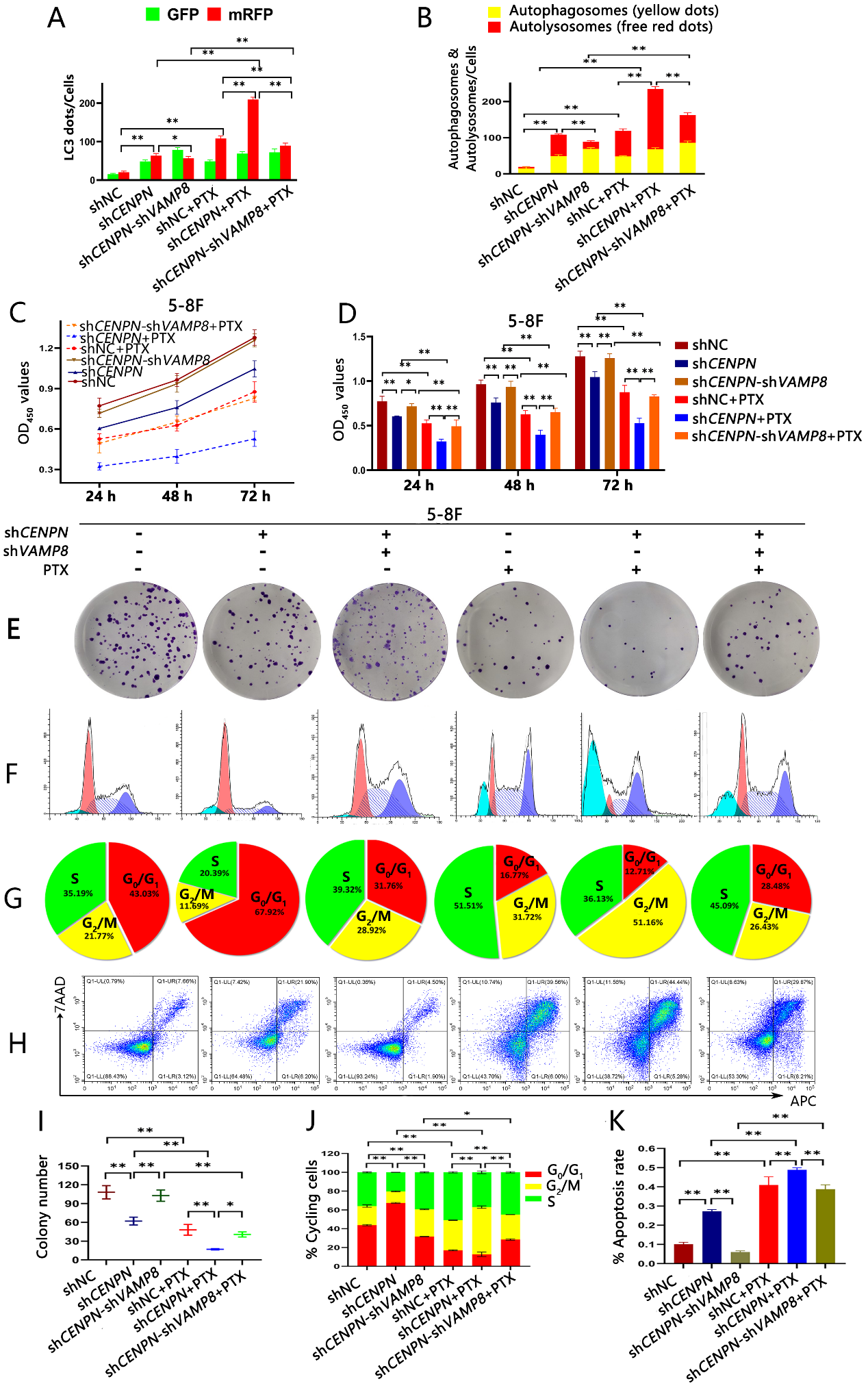


**Figure S8.** Sequential knockdown of *CENPN* and *VAMP8* reverses the effects of knockdown of *CENPN* alone on cell survival, clone formation, cell cycle progression, and apoptosis. (**A**, **B**) Bar chart showed the effect of PTX on autophagosomes and autolysosomes in 5-8F sh*CENPN*-sh*VAMP8* cells. (**C**, **D**) The CCK8 assay showed that sh*CENPN*-sh*VAMP8* cells had significantly increased viability compared with sh*CENPN* cells. (**E**, **I**) The clone formation assay showed that the proliferation ability of sh*CENPN*-sh*VAMP8* cells was significantly increased compared with that of sh*CENPN* cells. (**F**, **G**, **J**) Flow cytometry showed that sh*CENPN*-sh*VAMP8* cells had a lower percentage of cell cycle arrest than sh*CENPN* cells. (**H**, **K**) The flow cytometric apoptosis assay showed that sh*CENPN*-sh*VAMP8* cells had a lower apoptosis rate than sh*CENPN* cells. The concentration of PTX was 10 nM. Data are presented as mean ± SD. *, P<0.05. **, P<0.01.


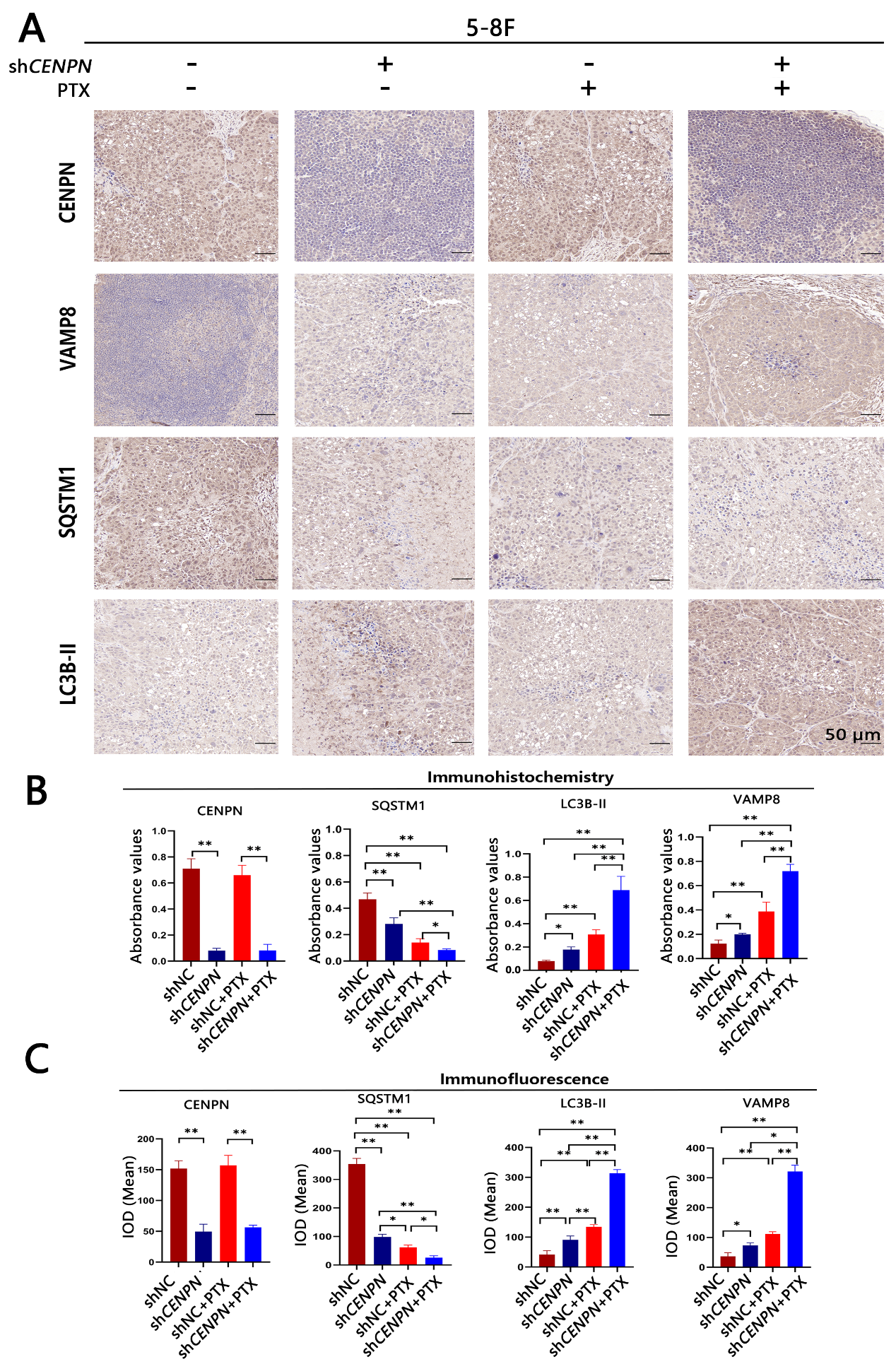


**Figure S9.** Knockdown of *CENPN* affects the levels of autophagy in NPC xenografts. (**A**) Representative immunohistochemical images showing autophagy-related protein levels in xenografts in each group (200×). (**B**) Comparison of the average absorbance values of autophagy in xenografts in each group, as determined by immunohistochemical staining. (**C**) Comparison of the IOD (mean) values of autophagy in xenografts in each group, as determined by immunofluorescence staining. Data are presented as mean ± SD. *, P < 0.05. **, P < 0.01.

**
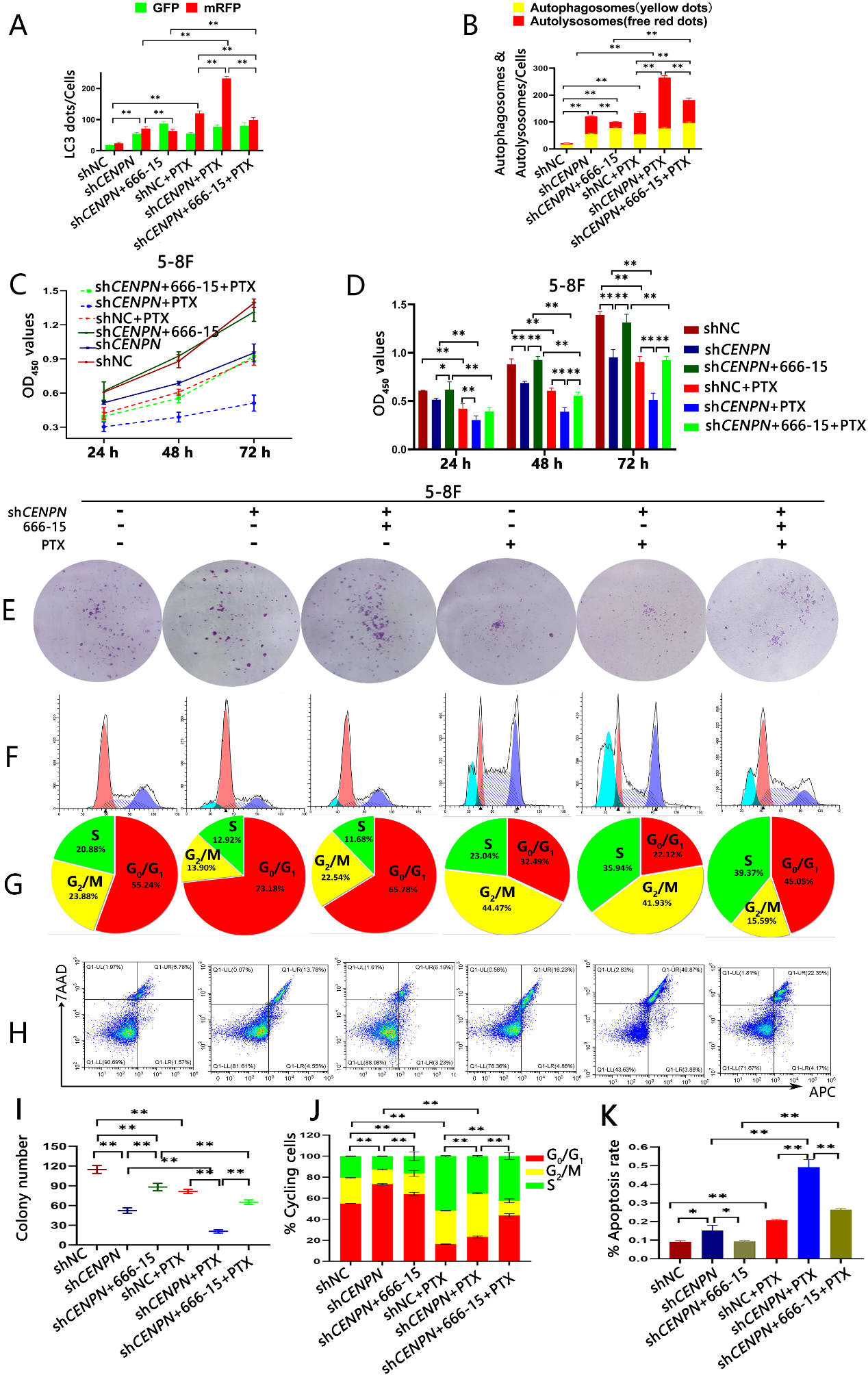
**

**Figure S10.** 666-15 inhibited the effect of *CENPN* knockdown on NPC cell survival, colony formation, cell cycle progression and apoptosis. (**A, B**) Bar chart showed the effect of 666-15 on autophagosomes and autolysosomes in 5-8F sh*CENPN* cells. (**C**, **D**) The CCK8 assay showed that 666-15 increased the viability and paclitaxel tolerance of 5-8F sh*CENPN* cells. (**E**, **I**) The colony formation assay showed that 666-15 could enhance the proliferation ability and paclitaxel tolerance of 5-8F sh*CENPN* cells. (**F**, **G**, **J**) Flow cytometry showed that 666-15 suppressed cell cycle arrest and decreased paclitaxel sensitivity in 5-8Fsh*CENPN* cells. (**H**, **K**) Flow cytometry showed that 666-15 reduced the apoptosis rate and paclitaxel sensitivity of 5-8F sh*CENPN* cells. The concentrations of 666-15 were 1.2 μΜ. The concentrations of PTX were 10 nM. Data are presented as mean ± SD. *, P < 0.05. **, P < 0.01.
